# Genetic Architecture of Ear Fasciation in Maize (*Zea mays*) under QTL Scrutiny

**DOI:** 10.1371/journal.pone.0124543

**Published:** 2015-04-29

**Authors:** Pedro Mendes-Moreira, Mara L. Alves, Zlatko Satovic, João Pacheco dos Santos, João Nina Santos, João Cândido Souza, Silas E. Pêgo, Arnel R. Hallauer, Maria Carlota Vaz Patto

**Affiliations:** 1 Departamento de Ciências Agronómicas, Escola Superior Agrária de Coimbra, Instituto Politécnico de Coimbra, Coimbra, Portugal; 2 Instituto de Tecnologia Química e Biológica António Xavier, Universidade Nova de Lisboa, Oeiras, Portugal; 3 Faculty of Agriculture, Department of Seed Science and Technology, University of Zagreb, Zagreb, Croatia; 4 Departamento de Biologia/UFLA, Universidade Federal de Lavras, Lavras-MG, Brasil; 5 Fundação Bomfim, Braga, Portugal; 6 Department of Agronomy, Iowa State University, Ames, Iowa, United States of America; Temasek Life Sciences Laboratory, SINGAPORE

## Abstract

**Maize ear fasciation:**

Knowledge of the genes affecting maize ear inflorescence may lead to better grain yield modeling. Maize ear fasciation, defined as abnormal flattened ears with high kernel row number, is a quantitative trait widely present in Portuguese maize landraces.

**Material and Methods:**

Using a segregating population derived from an ear fasciation contrasting cross (consisting of 149 F_2:3_ families) we established a two location field trial using a complete randomized block design. Correlations and heritabilities for several ear fasciation-related traits and yield were determined. Quantitative Trait Loci (QTL) involved in the inheritance of those traits were identified and candidate genes for these QTL proposed.

**Results and Discussion:**

Ear fasciation broad-sense heritability was 0.73. Highly significant correlations were found between ear fasciation and some ear and cob diameters and row number traits. For the 23 yield and ear fasciation-related traits, 65 QTL were identified, out of which 11 were detected in both environments, while for the three principal components, five to six QTL were detected per environment. Detected QTL were distributed across 17 genomic regions and explained individually, 8.7% to 22.4% of the individual traits or principal components phenotypic variance. Several candidate genes for these QTL regions were proposed, such as *bearded-ear1*, *branched silkless1*, *compact plant1*, *ramosa2*, *ramosa3*, *tasselseed4* and *terminal ear1*. However, many QTL mapped to regions without known candidate genes, indicating potential chromosomal regions not yet targeted for maize ear traits selection.

**Conclusions:**

Portuguese maize germplasm represents a valuable source of genes or allelic variants for yield improvement and elucidation of the genetic basis of ear fasciation traits. Future studies should focus on fine mapping of the identified genomic regions with the aim of map-based cloning.

## Introduction

“Fasciation” derives from the Latin word *fascis*, meaning “bundle” and is a reflection of increased cell proliferation [1]. One of the earliest reports of fasciation in maize dates from 1912 and, at that time, fasciated maize ears were frequently found in US maize fields [2], mostly in dent and pop maize germplasm [3]. There is a widespread occurrence of fasciated variants among vascular plants, and it has been reported that it increases crop yields [3]. Meristematic activity in the inflorescence has a profound influence on grain yield. Grain yield in maize is a complex, continuous trait that might be modified by a large number of genes including those controlling ear architecture traits. A thorough knowledge of the genes affecting these various components would lead to better yield modeling.

Many forward genetic screens have uncovered strong loss-of-function mutants with several altered maize ear architecture types and their responsible genes [4]. Examples are the *fasciated ear2* (*fea2*) [5,6], *compact plant2* (*ct2*) [7], *double cob1* (*dbcb1*) [8] and the *ramosa* genes (*ra1*, *ra2*, and *ra3*) [9].

While these mutants are useful for uncovering the normal function of genes, they rarely provide useful material for breeding efforts because they frequently display negative pleiotropic traits [4]. For example, the *fea2*, characterized by an increased kernel row number, is associated with a decrease in the length of the ear, as well as disorganized seed rows that limit the number of seeds per ear and their harvesting ability [5]. Additionally, much of the natural variation in inflorescence shape observed in maize is due to the cumulative effect of several loci. The responsible genes for the milder quantitative variation in these ear architecture traits or weaker alleles of these strong mutant variants will be particularly suitable for direct use on yield breeding approaches through Marker Assisted Selection (MAS). Many maize QTL studies have focused on ear architecture traits, with special interest on kernel row number [10–15] but just a few have attained fine mapping of major detected QTL [6,16–18]. Bommert et al. [6] have in fact isolated a weak allele of the *fea2* and showed that this allele increases kernel row number and number of kernels per ear, without causing a fasciated or shorter ear.

Since maize introduction to the country in the 15^th^ century, after Columbus, the importance of maize ear fasciation was quickly understood by Portuguese farmers who saw it as a way to improve production [19]. In traditional Portuguese maize landraces, ears are often found abnormally flattened and wider than normal, sometimes with irregular seed rows, but not particularly short in length. In addition to robustness and yield stability, Portuguese farmers preferred to select for large size ears without regard to shape, maintaining a certain level of diversity. This ear trait phenotype, known as “bear’s foot” in English, [20], corresponds to several popular names in Portuguese (“pé-de-porco”, “pata de porco”,”unha-de-porco”, “mão de morto”, “milho espalmado”, “mãozeira” or “milho das mãozinhas”), highlighting the importance of this trait for Portuguese farmers.

Contrary to other domestication and crop improvement traits [21], diversity in this ear trait was preferred and maintained by Portuguese farmers as an important parameter influencing yield [22]. In fact, fasciation trait expression varies with the environment, *i*.*e*., more inputs induce higher fasciation expression (for example, lower plant densities and more available nutrients) [3,23]. During the Portuguese maize collecting expedition in 2005 [22], 56% of the traditional maize landraces collected had some degree of fasciation, versus the 10% observed during the previous collecting missions in the 1980s. This fact indicates farmers’ preferences related to adaptation of their traditional agricultural systems: *i*.*e*., they selected a germplasm with enough yield plasticity and wider adaptability to different crop systems. Because of this finding, we might consider Portuguese maize landraces as a diversifying germplasm extremely important to seek novel alleles for breeding. Indeed the ear fasciation trait has already been exploited in a maize Portuguese Participatory Plant Breeding (PPB) program where as much as 18 up to 22 kernel rows per ear were obtained in the improved Open Pollinated Varieties (OPV) (e.g. ‘Pigarro’ or ‘Fandango’).

Early genetic studies of six Portuguese maize traditional landraces with a high frequency of abnormal ear shape (high level of fasciation expression) crossed with *ramosa* mutants indicated that fasciation was not associated with the *ramosa* genes (*ra1*, *ra2* or *ra3*) and a complex system of inheritance was proposed [24]. Additionally, this typical Portuguese ear fasciation was considered as a useful trait for improving yield when intermediate expression was attained, in order to allow certain uniformity in the ear and the plant [25]. Unfortunately, the genetic control of this ear trait was not elucidated, decreasing the possibilities of using it in an efficient and fast maize breeding approach.

The development of molecular markers allows us to study the genetic basis of complex quantitative traits, such as the typical Portuguese ear fasciation, in further detail and to develop tools for sustaining modern breeding approaches.

In the present study we: (1) determine the genetic relationships between a comprehensive set of ear architecture traits related with fasciation in a segregating F_2:3_ population, developed from a cross between contrasting (non-fasciated PB260 x fasciated PB266) inbred lines selected in Portugal, (2) identify chromosomal positions, size and effects of QTL involved in the inheritance of those traits, across two environments, using univariate and multivariate approaches and (3) identify possible candidate genes associated with these QTL.

## Results

### Genetic variation, heritabilities and phenotypic correlations

The parental accession PB266 had an average phenotypic value, for ear diameter 3 and 4, row number 2, cob/ear weight per ear and cob diameter 4, significantly higher than the parental accession PB260. Additionally, PB266 was also significantly more fasciated than PB260 (respectively 2.38 *versus* 1.41, near double) (Tables 1 and 2).

**Table 1:** Traits measured, codes and respective description of measurements.

Trait #	Codes	Name	Units/Scale	Data/plot	Measurement description
1	yld	Yield	Mg ha-1	1	Grain yield 15% moisture (Mg ha-1) = total Ear weight per ha x cwew x (100%—% moisture at harvest)/(100%-15%moisture) Grain moisture measured with the FARMPOINT moisture meter using amixed sample of four shelled ears grain.
2	cwew	Cob/ear weight at harvest	%	1	Ratio of cob weight in the ear weight per plot (sample of 4 ears)
3	en	Ears number	n°	1	Number of ears per square meter
4	av_ew	Ear weight at harvest	G	1	Average ear weight corrected for 15% moisture
5	l	Ear length	Cm	5	Ear length
6–7.	ed1, ed3	Ear diameter 1 and 3	Cm	5	Large diameter in the 1/3 bottom and top of the ear respectively
8–9.	ed2, ed4	Ear diameter 2 and 4	Cm	5	Small diameter in the 1/3 bottom and top of the ear respectively (90° rotation from large diameter)
10–11	cd1, cd3	Cob diameter 1 and 3	Cm	5	cd1 and 3 measure in the same way for ed’s
12–13	cd2, cd4	Cob diameter 2 and 4	Cm	5	cd2 and 4 measure in the same way for ed’s
14	kd	Kernel dept	Cm	5	Kernel dept, one kernel in the middle of the ear
15–16	m1, m2	Medulla 1 and 2	Cm	5	Large and small length of medulla, respectively, cob is cut in the Diameter 1 position [78,79]
17–18	rq1, rq2	Rachis 1 and 2	Cm	5	Large and small length of rachis; cob is cut in the Diameter 1 position [78,79]
19	ew	Ear weight	G	5	Ear weight, adjusted to 15% of grain moisture
20	cw	Cob weight	G	5	Cob weight, adjusted to 15% grain moisture
21	sw	Thousand-kernel weight	G	5	Thousand-kernel weight at 15% grain moisture
22	kw	Kernel weight	G	5	kernel weight per ear, adjusted to 15% grain moisture
23	e_cwew	Cob/ear weight per ear	%	5	Percentage of cob weight in the ear weight measured per ear at lab
24–25	r1, r2	Kernel-row number 1 and 2	n°	5	Row number in the 1/3 bottom and top of the ear respectively
26	fa	Fasciation	1 to 9	5	Fasciation degree (1—without fasciation and 9 as a maximum of fasciation)
27	cv	Ear convulsion	0 to 5	5	Convulsion intensity, kernel-row arrangement in the ear (0—without convulsion, regular kernel-row arrangement, 5—maximum of convulsion, without kernel-row arrangement)
28	kn	Kernel number	n°	5	Kernel number per ear
29	kr	Kernel per row	n°	5	Kernel number per row

**Table 2:** Phenotypic values (mean ± standard deviation) of the parental inbred lines PB260 and PB266, respective F_2:3_ families and quantitative genetic parameters for ear fasciation and related traits.

Trait	PB260^a^	PB266		F_2:3_ PB260 x PB266									
	c^b^	c	Sheffe^c^	c	m	c&m	c	m	c&m	ANOVA^f^			
	χ ± sd	χ ± sd		χ ± sd; KS^d^	χ ± sd; KS	r^e^	h^2^	h^2^	h^2^	G	E	REP(E)	G x E
**yld**	0.53	0.41	^ ^	3.9±1.24	4.04±1	0.44***	0.67	0.48	0.61	***	ns	ns	*
**cwew**			^ ^	0.26±0.06**	0.29±0.04*	0.22*	0.54	0.31	0.24	***	ns	***	**
**en**			^ ^	4.44±0.98**	4.62±0.84**	0.35***	0.66	0.37	0.51	***	ns	ns	**
**av_ew**			^ ^	82.0±16.0	87.0±19.5	0.29**	0.50	0.42	0.42	***	ns	*	*
**l**	11.24±1.57	9.24±2.11	PB266^a^<minF_2_ ^a^ ^b^<PB260^a^ ^b^<MaxF_2_ ^b^	13.16±1.67	14.14±1.6	0.71***	0.79	0.73	0.72	***	*	ns	ns
**ed1**	3.59±0.21	3.87±0.64	PB260^a^<minF_2_ ^a^<PB266^a^<MaxF_2_ ^b^	4.72±0.32	4.81±0.31	0.67***	0.76	0.71	0.78	***	ns	***	*
**ed3**	3.1±0.29	3.88±0.76	PB260^a^<minF_2_ ^a^<PB266^b^<MaxF_2_ ^c^	4.43±0.32	4.38±0.35	0.63***	0.81	0.63	0.76	***	ns	**	*
**ed2**	3.56±0.39	3.44±0.55	minF_2_ ^a^<PB266^a^<PB260^a^<MaxF_2_ ^b^	4.47±0.33	4.58±0.3	0.65***	0.75	0.68	0.75	***	ns	***	*
**ed4**	3.04±0.37	3.58±0.73	PB260^a^<minF_2_ ^a^ ^b^<PB266^b^<MaxF_2_ ^c^	4.26±0.3	4.2±0.31	0.54***	0.79	0.53	0.69	***	ns	**	*
**r1**	10.24±2.54	10.94±3.87	PB260^a^<PB266^a^<minF_2_ ^b^<MaxF_2_ ^c^	16.6±1.57	16.83±1.73	0.7***	0.76	0.77	0.81	***	ns	ns	*
**r2**	9.65±2	13.5±4.94	PB260^a^< minF_2_ ^a^ ^b^<PB266^b^< MaxF_2_ ^c^	16.67±1.6	16.72±1.85	0.72***	0.79	0.78	0.83	***	ns	ns	*
**fa**	1.41±0.51	2.38±0.62	minF_2_ ^a^<PB260^b^<PB266^c^<MaxF_2_ ^d^	2.41±0.67**	2.09±0.6**	0.67***	0.61	0.70	0.73	***	ns	***	ns
**cv**	2.59±0.71	2.44±1.09	minF_2_ ^a^<PB266^b^<PB260^b^<MaxF_2_ ^c^	2.31±0.4*	2.21±0.36**	0.44***	0.49	0.35	0.59	***	ns	***	ns
**kd**	0.73±0.1	0.88±0.1	PB260^a^<PB266^a^ ^b^<minF_2_ ^a^ ^b^<MaxF_2_ ^b^	1.02±0.06*	1±0.05	0.64***	0.84	0.65	0.75	***	*	ns	**
**ew**	47.54±16.23	46.12±24.11	PB266^a^<PB260^a^<minF_2_ ^a^<MaxF_2_ ^b^	127.04±25.12	133.07±23.21	0.53***	0.72	0.56	0.68	***	ns	**	*
**kw**	37.06±14.87	33.02±19.18	PB266^a^<PB260^a^<minF_2_ ^a^<MaxF_2_ ^b^	100.53±19.36	102.32±17.24	0.52***	0.71	0.51	0.68	***	ns	**	*
**cw**	10.42±3.61	12.89±6.86	minF_2_ ^a^<PB260^a^<PB266^a^<MaxF_2_ ^b^	26.51±7.15	30.75±7.21	0.64***	0.75	0.73	0.68	***	*	ns	*
**e_cwew**	0.23±0.09	0.31±0.15	minF_2_ ^a^<PB260^b^<PB266^c^<MaxF_2_ ^c^	0.21±0.03	0.23±0.03	0.77***	0.73	0.82	0.69	***	*	ns	ns
**kn**	151.51±54.36	139.68±35.65	PB266^a^<PB260^a^<minF_2_ ^a^<MaxF_2_ ^b^	353.61±68.86	389.68±60.12	0.59***	0.76	0.52	0.64	***	ns	**	ns
**sw**	244.3±43.78	226.74±100.4	minF_2_ ^a^<PB266^b^<PB260^b^<MaxF_2_ ^c^	288.06±30.72	264.04±28.48	0.64***	0.81	0.67	0.58	***	***	ns	*
**kr**	15.18±3.81	12.38±3.61	PB266^a^<minF_2_ ^a^<PB260^a^<MaxF_2_ ^b^	23.36±3.49	25.76±3.02	0.68***	0.78	0.67	0.64	***	*	ns	ns
**cd1**	2.72±0.36	2.7±0.47	minF_2_ ^a^<PB266^a^<PB260^a^<MaxF_2_ ^b^	3.2±0.26	3.33±0.26	0.73***	0.72	0.79	0.77	***	ns	**	ns
**cd3**	2.1±0.19	2.51±0.5	minF_2_ ^a^<PB260^a^<PB266^a^<MaxF_2_ ^b^	2.82±0.23	2.77±0.27	0.68***	0.80	0.71	0.79	***	ns	*	*
**cd2**	2.56±0.4	2.4±0.44	minF_2_ ^a^<PB266^a^ ^b^<PB260^b^<MaxF_2_ ^c^	2.91±0.23	3.04±0.21*	0.68***	0.69	0.71	0.71	***	ns	***	ns
**cd4**	2.01±0.21	2.33±0.44	minF_2_ ^a^<PB260^a^<PB266^b^<MaxF_2_ ^c^	2.62±0.19	2.59±0.21	0.53***	0.74	0.57	0.69	***	ns	*	*
**m1**	0.73±0.2	0.66±0.36	PB266^a^<PB260^a^<minF_2_ ^a^<MaxF_2_ ^b^	1.32±0.17	1.39±0.21	0.67***	0.64	0.76	0.76	***	ns	*	ns
**m2**	0.62±0.19	0.5±0.26	PB266^a^<minF_2_ ^a^<PB260^a^<MaxF_2_ ^b^	1.02±0.14	1.06±0.16	0.67***	0.69	0.60	0.78	***	ns	*	ns
**rq1**	1.89±0.18	1.8±0.32	minF_2_ ^a^<PB266^a^<PB260^a^<MaxF_2_ ^b^	2.32±0.23	2.46±0.23	0.67***	0.72	0.79	0.68	***	ns	***	**
**rq2**	1.62±0.2	1.54±0.38	minF_2_ ^a^<PB266^a^ ^b^<PB260^b^<MaxF_2_ ^c^	1.97±0.21	2.11±0.2	0.66***	0.72	0.68	0.67	***	ns	***	ns

^a^ PB260 and PB266 data obtained from 2010 and 2012 organic production field trial, with 2 replications at Coimbra. F_2:3_ data obtained from two conventional field trials at Coimbra and Montemor, with two replications.

^b^ c—Coimbra and m—Montemor

^c^ minF_2_—top five minimum values of F_2_ PB260 x PB266; MaxF_2_—top five maximum values of F_2:3_ PB260 x PB266. Significant differences exist when no letter repetition occurred between groups

^d^ KS—*, **—Significance of the Kolmogorov-Smirnov's test of normality

^e^ r—correlation between the two environments per trait. h^2^—broad sense heritabilities

^f^ Significance of the sources of variability: G—Genotype, E—Environment, Rep (E)—Repetitions within Environment, G x E—Genotype x Environment Interaction

Levels of significance: ns non-significant value

* significant at P < 0.05

** significant at P < 0.01

*** significant at P < 0.001

Traits measured: yld—yield; cwew—cob/ear weight at harvest; en—ears number; av_ew—20 ears average weight at harvest t; l—ear length; ed 1 to 4—ear diameter 1 to 4; cd1 to 4—cob diameter 1 to 3; kd—kernel dept; m1, m2—medulla 1 and 2; rq1, rq2-rachis 1 and 2; ew-ear weight; cw-cob weight; sw-thousand kernel weight; kw—kernel weight; e_cwew—cob/ear weight per ear; r1, r2—kernel-row number 1 and 2; fa—fasciation; cv—ear convulsion; kn—kernel number; kr—kernel per row.

Genotypic effects were highly significant for all investigated traits (P < 0.01). Nevertheless, genotype x environment interaction was significant for the majority of the traits, with the exception of ear length, fasciation, convulsion, cob/ear weight per ear, kernels per row, ear diameter 2, medulla 1 and 2 and rachis 2 (Tables 1 and 2). Because of these findings, and to better understand the stability of the QTL across multiple environments, QTL mapping was performed separately for each environment data set.

F_2:3_ families showed a large and continuous variation for the investigated traits. The Kolmogorov-Smirnov's test of normality showed that a few of the studied traits were not normally distributed, such as the fasciation trait (Tables 1 and 2). Transformation of fasciation data by the natural logarithm function improved normality, but had little effect on the analyses. Hence results were described only for the untransformed data.

In general, the phenotypic values of the two parental inbred lines were significantly different from at least one of the most extreme F_2_ plants (P ≤ 0.05), and transgressive segregation was observed for all the traits with the exception of kernel depth and ear length. However, these comparisons should be considered with caution, since the presented data from parental lines and F_2:3_ families were obtained in different environmental conditions (Table 2), during several years of evaluation, at least for the parental accessions.

Broad-sense heritabilities of investigated traits across both environments were in general above or equal to 0.70, apart from grain yield, the cob/ear weight at harvest, number of ears, average ear weight at harvest, ear convulsion, kernel number, thousand kernel weight and number of kernel rows. In general, broad sense heritabilities were higher at the Coimbra than Montemor environment with the exception of rachis 1, medulla 1, cob diameters 1 and 2, cob/ear weight per ear, kernel row numbers and fasciation. Fasciation heritability was 0.73 across both environments and ranged from 0.61 at Coimbra to 0.70 at Montemor (Table 2).

Trait correlations between the two environments were highly significant (P < 0.001) with the exception of cob weight (P < 0.01) and rachis 2 (P < 0.05) (Table 2 and S1 Table). Correlation coefficients range from 0.22 to 0.77 (rachis 2 and thousand kernel weight, respectively), but were above 0.60 for many traits, as in the case of fasciation (0.67) and ear diameter 3 (0.63). High correlations (0.70 to 1.00) were observed for ear length (0.71), row number 1 and 2 (0.70 and 0.71), cob/ear weight per ear (0.77) and cob diameter 1 (0.73) (Table 2).

The highest correlations (0.9–1.00) observed in both environments were among ear diameters from the base of the ear or from the top of the ear (ear diameters 1 and 2 and ear diameters 3 and 4, respectively), rows number 1 and 2 and cob diameter 1 and 2 with rachis 1 and 2, respectively. The rachis and medulla had high correlations, as was expected, because their differences represent the thickness obtained by glumes, paleas and lemmas components. In addition, ear and kernel weight were also highly correlated (S1 Table).

The fasciation trait at Coimbra and Montemor was correlated with ear diameter 3 (0.53 and 0.76), row number 2 (0.45 and 0.75) and cob diameter 3 (0.59 and 0.79). These two last traits presented the highest correlations between ear diameters and row numbers. Additionally, correlations varying between 0.70–0.90 were detected for both environments for medulla 1 and 2 and rachis 1 and 2, and also among and within ear diameters (ear diameters 1 and 2 with ears diameters 3 and 4) and cob diameters (cob diameters 1 with 2 and 3, and cob diameter 3 with 4). High correlations among rachis 1 and cob diameter 2 and medulla 1 were also observed. The yield and cob weight were correlated with ear and kernel weight. Finally, kernel number was correlated with ear and kernel weight and number of kernels per row. This last trait was also correlated with ear length (S1 Table).

The three principal components explained 73.95% and 71.1% of the variation, respectively, at the Coimbra and Montemor environments. Principal component 1 (PC1) for Coimbra explained 43.61% of the total variation present in the data set. For this PC1, the traits that contribute the most for explaining variation were ear diameter 1 and 2 (correlation, 0.90 to 1.00) and ear diameter 3 and 4, ear, kernel and cob weight, cob diameters, medulla and rachis correlation, 0.70 to 0.90). The principal component 2 explained 17.6% of the total variation present in this Coimbra data set. For this PC2, the traits that contribute the most for explaining variation were grain yield, ear number, ear length and number of kernel per row. The principal component 3 explained, in Coimbra, 12.7% of the total variation present in the data set. For this PC3, the trait contributing the most for explaining variation was the cob/ear weight per ear (S2 Table).

For Montemor, the principal component 1 explained 42.93% of the total variation present in the data set. For this PC1, the traits that contribute the most for the explaining variation were ear diameter 1 and 2 (correlation 0.90 to 1.00) and ear diameter 3 and 4, ear and kernel weight, cob diameters, medulla 1 and rachis (correlation, 0.70 to 0.90). The principal component 2 explained 17.09% of the total variation present in the Montemor data set. For this PC2, the trait contributing the most for the explaining variation was ear length. The principal component 3 explained, in Montemor, 11.06% of the total variation present in the data set (S2 Table).

### Map of the PB260 x PB266 progeny

The 17 AFLP primer combinations selected had a total of 451 dominantly scored polymorphic fragments on the F_2_ population, with an average of 27 polymorphic fragments per primer combination, ranging from 18 to 35, respectively, in the primer combinations E36-M49/E36-M50 and E32-M47. Among these 451, 227 were specific from PB260 and 224 were PB266 specific. In addition to the AFLP, 60 selected SSR markers were codominantly scored on the F_2_ population.

From the original molecular data set (149 F_2_ individuals screened with 511 markers—60 SSR and 451 AFLP polymorphic markers), we removed the 23 individuals and 57 markers with more than 10% missing values, plus 36 markers with a severe segregation distortion (P≤ 0.05) and 167 redundant markers clustered at the same position.

After performing a preliminary map analysis, three more individuals were removed due to their very improbable genotypes (singletons), as well as three more markers presenting suspected linkages with other markers.

Based on the remaining genotypic data of 248 markers screened on 123 F_2_ individuals, 10 linkage groups were obtained. Fifty-four markers were not assigned to any of the 10 resulting linkage groups. A linkage map containing 194 markers (144 dominant and 50 codominant) was developed, covering a total map distance of 1172.5 cM, with an average distance of approximately 6 cM per marker (Table 3).

**Table 3:** Refined genetic linkage map of the maize population F_2_ (PB260xPB266) (used for QTL mapping).

Linkage Group	No. Markers	No. Codominant Markers	χ2 mean	Length (cM)	Average distance (cM)
1	29	6	1.115	167.63	5.78
2	27	7	1.060	146.00	5.41
3	19	5	0.924	145.16	7.64
4	22	3	0.792	107.96	4.91
5	18	6	0.598	152.78	8.49
6	14	4	0.724	85.72	6.12
7	16	5	1.351	93.45	5.84
8	16	5	1.098	90.17	5.64
9	16	4	0.913	75.67	4.73
10	17	5	1.141	107.93	6.35
Average	19.4	5	-	117.2	6.09
Total	194	50	-	1172.5	-

Inspection of the individual linkage group χ^2^ values gave insights into the reliability of the obtained map. The χ^2^ values of the majority of the linkage groups were ≤ 1 except for linkage groups 1, 7 and 10, varying from 1.115 to 1.351 (Table 3). Given the high densities of markers, these χ^2^ values indicated that the map was relatively reliable. This map was then used for QTL identification.

### QTL detected on the PB260 x PB266 progeny

#### Single trait QTL analysis

**Fig 1:**
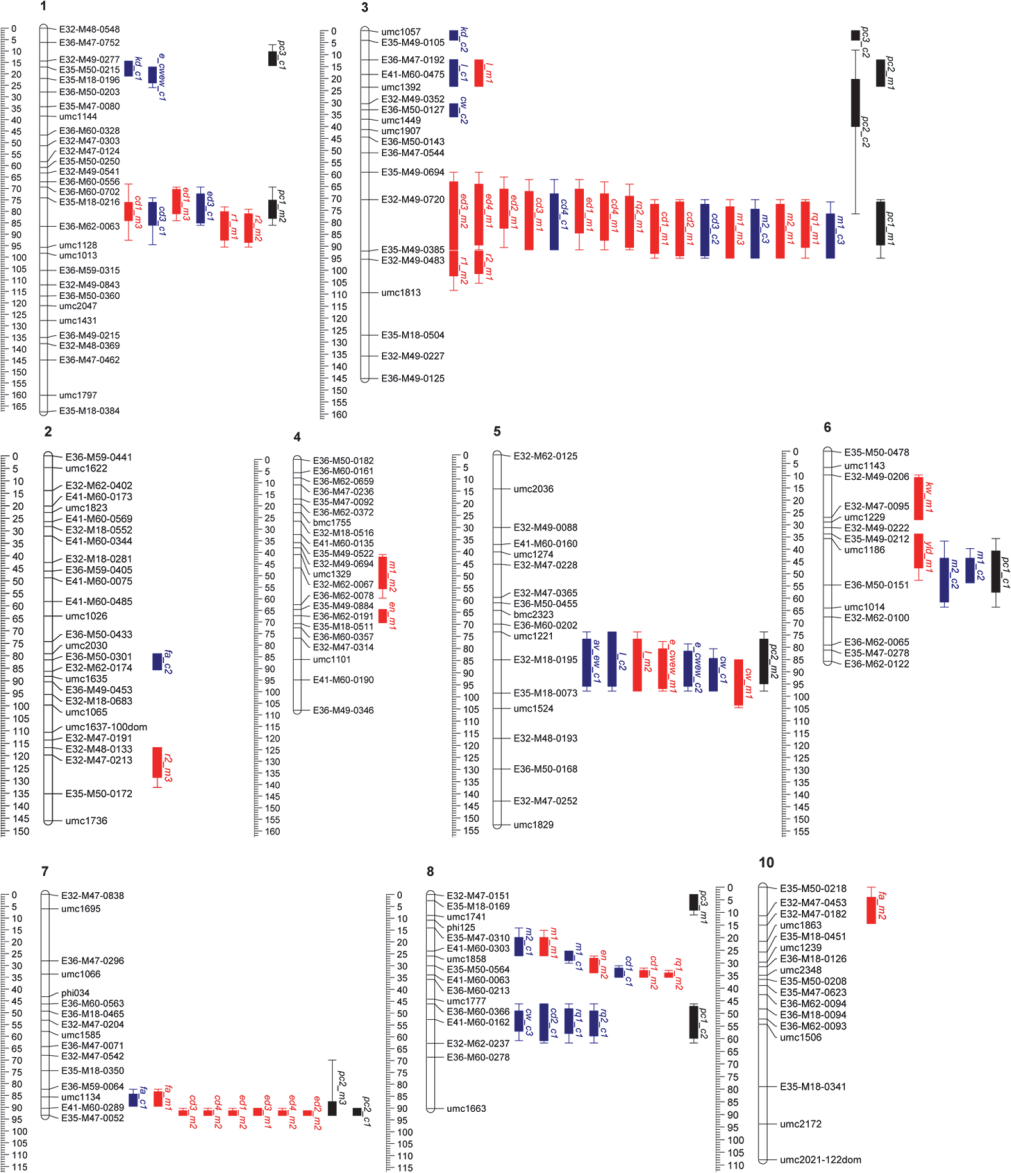
QTL detected for ear fasciation and related traits using 149 F_2:3_ families (PB260 (non-fasciated) x PB266 (fasciated)) at two environments in Portugal (Coimbra and Montemor). Bar positions indicate the locations of quantitative trait loci (QTL): outer and inner interval correspond to 1-LOD and 2-LOD support interval, and are indicated as full box and a single line respectively. QTL nomenclature was arranged by the trait name plus environment abbreviation (c = Coimbra and m = Montemor) and the order number of detected QTL for the same trait in the genome (the higher the n°, the lower the contribution of the detected QTL for the explained phenotypic variability).

**Fig 2:**
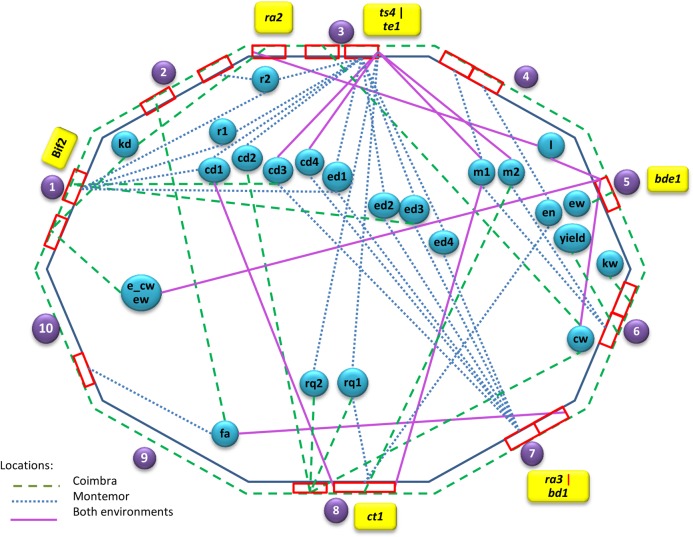
Representation of the ear fasciation and related traits QTL regions per maize chromosome, with indication of respective detection environment. For each chromosomal region, the respective candidate genes are indicated.

QTL were detected for the majority of the 29 traits with the exception of cob/ear weight at harvest, ear convulsion, kernel number, thousand kernel weight, kernels per row and ear weight at harvest. Sixty-five QTL (26 at the Coimbra environment and 39 at the Montemor environment), summarized in 17 different regions, were detected for 23 traits (Table 4). Eleven of these QTL were detected in both environments (constitutive) and distributed across four chromosomes (3, 5, 7 and 8). Strong clustering of QTL (with colocalized QTL for 3 or more traits) was observed in seven regions (Fig 1, Fig 2).

**Table 4:** Quantitative Trait Loci for fasciation and related ear traits, estimated from 149 F_2:3_ maize families (PB260 x PB266).

Trait	Environment^a^	QTL	Chromosome	QTL Position (cM)^b^	Flanking markers^c^	Peak LOD Score	Additive effect^d^	Gene Action^e^	R^2^ ^f^
Yield	M	yld_m1	6	35.68	umc1186	4.12	-0.52	A	14.3
Ears number	M	en_m1	4	68.29	E36-M62-0191 / E35-M18-0511	5.34	0.90	PD	16.7
	M	en_m2	8	29.96	E35-M50-0564	3.97	-0.84	A	12.1
Ear lenght	C	l_c1	3	23.26	E41-M60-0475 / umc1392	5.69	0.86	PD	17.1
	C	l_c2	5	84.60	E32-M18-0195	3.83	-0.93	PD	11.2
	M	l_m1	3	15.24	E36-M47-0192 / E41-M60-0475	4.04	0.70	D	12.5
	M	l_m2	5	87.60	E32-M18-0195 / E35-M18-0073	3.61	-0.91	PD	11.1
Ear diameter 1	M	ed1_m1	3	74.47	E32-M49-0720 / E35-M49-0385	7.11	-0.25	PD	18.4
	M	ed1_m2	7	93.41	E41-M60-0289 / E35-M47-0052	4.50	0.09	OD	11.2
	M	ed1_m3	1	77.11	E35-M18-0216 / E36-M62-0063	3.62	-0.07	OD	8.7
Ear diameter 3	C	ed3_c1	1	79.11	E35-M18-0216 / E36-M62-0063	4.13	-0.20	PD	14.3
	M	ed3_m1	7	93.41	E41-M60-0289 / E35-M47-0052	5.64	0.11	OD	16.4
	M	ed3_m2	3	74.47	E32-M49-0720 / E35-M49-0385	5.04	-0.25	PD	14.4
Ear diameter 2	M	ed2_m1	3	73.47	E32-M49-0720 / E35-M49-0385	6.46	-0.23	D	19.1
	M	ed2_m2	7	93.45	E35-M47-0052	3.84	0.07	OD	10.9
Ear diameter 4	M	ed4_m1	3	74.47	E32-M49-0720 / E35-M49-0385	4.83	-0.21	PD	14.2
	M	ed4_m2	7	93.45	E35-M47-0052	4.62	0.06	OD	13.6
Kernel-row number 1	M	r1_m1	1	83.11	E35-M18-0216 / E36-M62-0063	5.89	-0.97	D	17.5
	M	r1_m2	3	95.01	E35-M49-0385 / E32-M49-0483	4.64	-1.05	PD	13.6
Kernel-row number 2	M	r2_m1	3	94.01	E35-M49-0385 / E32-M49-0483	6.64	-1.27	PD	17.2
	M	r2_m2	1	89.57	E36-M62-0063 / umc1128	6.50	-0.95	D	16.6
	M	r2_m3	2	121.65	E32-M47-0213 / E35-M50-0172	3.81	0.55	OD	9.3
Fasciation	C	fa_c1	7	86.57	umc1134 / E41-M60-0289	4.44	0.20	OD	13.2
	C	fa_c2	2	81.19	umc2030 / E36-M50-0301	3.89	0.31	PD	11.5
	M	fa_m1	7	87.57	umc1134 / E41-M60-0289	4.86	0.22	OD	14.1
	M	fa_m2	10	11.00	E35-M50-0218 / E32-M47-0453	4.22	0.30	PD	12.3
Kernel dept	C	kd_c1	1	19.02	E35-M50-0215 / E35-M18-0196	6.38	-0.04	A	18.5
	C	kd_c2	3	0.00	umc1057	4.81	-0.03	PD	13.6
Ear weight	C	ew_c1	5	84.60	E32-M18-0195	3.81	-15.09	PD	13.3
Kernel weight	M	kw_m1	6	21.69	E32-M49-0206 / E32-M47-0095	3.77	-9.43	A	13.2
Cob weight	C	cw_c1	5	91.60	E32-M18-0195 / E35-M18-0073	6.84	-4.86	A	16.8
	C	cw_c2	3	33.02	E36-M50-0127	4.31	3.28	PD	10.4
	C	cw_c3	8	52.64	E41-M60-0162	3.93	2.91	PD	9.4
	M	cw_m1	5	91.60	E32-M18-0195 / E35-M18-0073	4.88	-4.95	A	16.7
Cob/ear weight per ear	C	e_cwew_c1	1	21.02	E35-M50-0215 / E35-M18-0196	7.33	0.02	A	19.5
	C	e_cwew_c2	5	88.60	E32-M18-0195 / E35-M18-0073	5.99	-0.02	PD	15.3
	M	e_cwew_m1	5	87.60	E32-M18-0195 / E35-M18-0073	5.13	-0.02	PD	17.5
Cob diameter 1	C	cd1_c1	8	32.96	E35-M50-0564 / E41-M60-0063	5.38	0.16	PD	18.3
	m	cd1_m1	3	82.47	E32-M49-0720 / E35-M49-0385	5.84	-0.19	A	16.4
	M	cd1_m2	8	33.87	E41-M60-0063	5.45	0.15	PD	15.5
	M	cd1_m3	1	80.11	E35-M18-0216 / E36-M62-0063	3.78	-0.08	OD	9.0
Cob diameter 3	C	cd3_c1	1	81.11	E35-M18-0216 / E36-M62-0063	4.53	-0.13	D	13.8
	C	cd3_c2	3	83.47	E32-M49-0720 / E35-M49-0385	4.39	-0.14	PD	13.8
	M	cd3_m1	3	78.47	E32-M49-0720 / E35-M49-0385	6.30	-0.21	A	17.5
	M	cd3_m2	7	93.45	E35-M47-0052	6.09	0.10	OD	17.2
Cob diameter 2	C	cd2_c1	8	52.64	E41-M60-0162	3.77	0.12	PD	13.2
	m	cd2_m1	3	80.47	E32-M49-0720 / E35-M49-0385	5.07	-0.16	PD	17.3
Cob diameter 4	c	cd4_c1	3	79.47	E32-M49-0720 / E35-M49-0385	3.70	-0.13	PD	12.9
	m	cd4_m1	3	76.47	E32-M49-0720 / E35-M49-0385	6.29	-0.16	PD	18.2
	m	cd4_m2	7	93.45	E35-M47-0052	4.35	0.03	OD	12.3
Medulla 1	c	m1_c1	8	25.84	E41-M60-0303 / umc1858	7.10	0.09	PD	17.9
	c	m1_c2	6	51.68	umc1186 / E36-M50-0151	5.94	-0.10	D	13.3
	c	m1_c3	3	88.47	E32-M49-0720 / E35-M49-0385	4.01	-0.08	PD	9.5
	m	m1_m1	8	25.84	E41-M60-0303 / umc1858	6.26	0.11	PD	15.0
	m	m1_m2	4	47.66	E32-M62-0067 / E36-M62-0078	5.66	-0.12	A	13.7
	m	m1_m3	3	86.47	E32-M49-0720 / E35-M49-0385	4.71	-0.12	A	11.2
Medulla 2	c	m2_c1	8	23.84	E41-M60-0303	5.00	0.07	PD	12.8
	c	m2_c2	6	51.68	umc1186 / E36-M50-0151	4.43	-0.07	D	11.2
	c	m2_c3	3	86.47	E32-M49-0720 / E35-M49-0385	4.18	-0.07	PD	10.5
	m	m2_m1	3	82.47	E32-M49-0720 / E35-M49-0385	5.25	-0.12	A	17.8
Rachis 1	c	rq1_c1	8	52.64	E41-M60-0162	5.38	0.15	A	18.2
	m	rq1_m1	3	78.47	E32-M49-0720 / E35-M49-0385	5.77	-0.17	PD	16.5
	m	rq1_m2	8	34.87	E41-M60-0063 / E36-M60-0213	4.76	0.12	PD	13.7
Rachis 2	c	rq2_c1	8	52.64	E41-M60-0162	4.47	0.11	PD	15.4
	m	rq2_m1	3	79.47	E32-M49-0720 / E35-M49-0385	5.96	-0.15	PD	20.0

^a^ m = Montemor; c = Coimbra

^b^ QTL position in cM from the top of the chromosome

^c^ molecular markers flanking the support interval estimated at a LOD fall of -2.00

^d^ Additive effect = (phenotypic mean of the PB260 allele genotypes—phenotypic mean of the PB266 allele genotypes) / 2 [79]; negative values indicate that the PB266 allele increased trait additive value

^e^ Gene action: A—additive if |dominant effect/additive effect| < 0.2; PD—partial dominance 0.2 < |d/a| < 0.8, D—dominance 0.8 < |d/a| < 1.2; OD—overdominance |d/a| > 1.2

^f^ Percent explained phenotypic variance

Four QTL detected for the fasciation trait were localized, one in chromosome 2 (Coimbra), and another in chromosome 10 (Montemor); two were constitutive in chromosome 7.

The amount of explained phenotypic variance ranged from 11.5% to 14.1% in each individual QTL detected, and in total the fasciation QTL explained 24.7% to 26.4% of the phenotypic variance at Coimbra and Montemor respectively (Table 4, Fig 1) assuming the absence of epistasis. In these detected fasciation QTL, the alleles for increasing the trait were always contributed by the parental accession PB260 (Table 4), with a lower level of fasciation, which is in agreement with the detected transgressive segregation in the F_2_ population (Table 2).

A single QTL was detected for yield in chromosome 6, accounting for 14.3% of the total phenotypic variance, and only at Montemor, where PB266 contributed with the allele increasing yield.

Two QTL were detected for ear number, in chromosomes 4 and 8, accounting for 16.7% and 12.1% of the total phenotypic variance at Montemor, respectively. In this case, the increasing alleles were, in the QTL located in chromosome 8 (en_m2), provided by PB266 and, in the QTL located in chromosome 4 (en_m1), by PB260 (Table 4).

Two QTL for ear length were constitutively detected in chromosomes 3 and 5. QTL on chromosome 3 explained 17.1% and 12.5% of the phenotypic variance at Coimbra and Montemor, respectively. The QTL in chromosome 5 explained 11.1% and 11.2% of the phenotypic variance (Table 4, Fig 1). Per environment, considering the absence of epistasis, the detected QTL explained a total of 28.0% (Coimbra) to 23.6% (Montemor) of the phenotypic variance for ear length.

Also in this trait and in agreement with the transgressive segregation detected in the F_2_ population, increasing alleles were contributed by the two parental lines (PB266 in the QTL detected in chromosome 5 and PB260 in the QTL detected in chromosome 3) (Tables 2 and 4).

Twenty-three QTL involved in inheritance of ear and cob diameters were detected, with a maximum of three QTL detected per trait in each environment. Ear and cob diameter QTL were mainly detected in chromosomes 1, 3 and 7, with chromosome 8 involved in cob diameter inheritance. QTL for cob diameter 1, in chromosome 8, and cob diameters 3 and 4, in chromosome 3, were constitutively detected.

The percentage of explained phenotypic variance per individual ear diameter QTL ranged from 8.7% in chromosome 1 (ear diameter 1 at Montemor) to 19.1% in chromosome 3 (ear diameter 2 at Montemor). For individual cob diameters QTL, the percentage of explained phenotypic variance ranged from 9.0% in chromosome 1 (Montemor) to 18.3% in chromosome 8 (Coimbra) (Table 4, Fig 1). Per environment, the total of explained phenotypic variance per ear diameters, considering the absence of epistasis, ranged from 14.3% (Coimbra, ear diameter 3) to 38.3% (Montemor, ear diameter 1) and per cob diameters from 12.9% (Coimbra, cob diameter 4) to 40.9% (Montemor, cob diameter 1). For cob and ear diameters QTL, 14 alleles increasing (chromosome 1 and 3, with 4 and 10 QTL respectively) and 9 alleles decreasing the traits (chromosome 7 and 8, with 6 and 3 QTL respectively; with chromosome 7 with QTL only detected at Montemor) were contributed by the parental accession PB266 (Table 2, Table 4).

QTL for row number 1 and 2 were only detected at one environment (Montemor) and included two colocalized QTL in chromosomes 1 and 3, for row number 1 and 2, and an additional QTL in chromosome 2, for row number 2. Individual QTL explained 13.6% to 17.5% of total phenotypic variance of kernel row number 1 (in total 31.1%, considering the absence of epistasis) and 9.3% to 17.2% in kernel row number 2 (in total 43.1%, considering the absence of epistasis) (Table 4, Fig 1).

Four alleles for increasing the trait phenotypes (chromosome 1 and 3, with 2 QTL each) and one allele for decreasing (chromosome 2, exclusively for row number 2) were contributed by the parental accession PB266 (Table 2 and Table 4).

Two QTL for kernel depth were detected in chromosome 1 and 3, only at one environment (Coimbra), explaining 13.6% to 18.5% of the total phenotypic variance, respectively. Increasing alleles were always contributed by PB266 (Table 4, Fig 1).

One QTL was detected for ear weight, only at Coimbra (explaining 13.3% of the phenotypic variance), in chromosome 5, where the increasing alleles were contributed by PB266. Also only one QTL was detected for kernel weight, at environment Montemor, in chromosome 6 (explaining 13.2% of the phenotypic variance) with the increasing allele being contributed by PB266 (Table 4, Fig 1).

One to three QTL were identified at Montemor and Coimbra, respectively, for cob weight in chromosome 3, 5 and 8. QTL detected in chromosome 5 were constitutive. Individual QTL explained 9.4% to 16.8% of the phenotypic variance and in total, per environment, explained 16.7% (Montemor) to 36.6% (Coimbra) of total phenotypic variance (Table 4, Fig 1). Both PB260 and PB266 contributed with alleles for increasing cob weight (Table 2).

For the cob/ear weight per ear, one to two QTL were detected per environment, one constitutive in chromosome 5 and another only detected in Coimbra (chromosome 1). The constitutive QTL explained, respectively, 17.5% and 15.3% of the phenotypic variance in Coimbra and Montemor and the QTL at chromosome 1 for Coimbra, 19.5% of the phenotypic variance (Table 4, Fig 1). PB266 contributed with the increasing allele of the constitutive QTL detected in chromosome 5 and PB260 with the increasing allele of the QTL detected in chromosome 1 (Table 4) (Table 2). In total, and per environment, the detected QTL explained 17.5% (Montemor) to 34.8% of total phenotypic variance of the cob/ear weight per ear.

For medulla 1 and 2, one to three QTL were detected, per environment, in chromosome 3, 4, 6 and 8. Two QTL for medulla 1 and 2 were constitutive, located, respectively, in chromosome 3 and 8. Individual QTL effects varied from 9.5% to 17.9% of explained variability. In total, and per environment, the detected QTL explained 39.9% (Montemor) to 40.7% (Coimbra) of the medulla 1 phenotypic variance, and 17.8% (Montemor) to 34.5% (Coimbra) of medulla 2, (Table 4, Fig 1).

For medulla QTL, 7 alleles for increasing the trait phenotypes (chromosome 3, 4 and 6, with 4, 1 and 2 QTL respectively) and 3 alleles for decreasing (chromosome 8) were contributed by the parental accession PB266, (Table 2 and Table 4).

For rachis 1 and 2, one to two QTL were detected, per environment, in chromosome 3 and 8, but none of these QTL were constitutive. They explained a total of 18.2% (Coimbra) to 30.2% (Montemor) of the phenotypic variance of rachis 1 and a total of 15.4% (Coimbra) to 20.0% (Montemor) of rachis 2, considering the absence of epistasis (Table 4, Fig 1).

For rachis 1 and 2 QTL, alleles for increasing and for decreasing the traits were contributed by the parental accession PB266, (Tables 2 and 6, Fig 1).

#### QTL analysis using principal components (PC)

First three principal components accounting for 73% and 71% of variation in Caldeirão and Montemor, respectively, were used to map QTL associated with maize ear architecture (Table 5, Fig 1).

**Table 5:** Quantitative Trait Loci for the first three PCs, derived from 29 traits in the F_2:3_ maize families (PB260xPB266) in two environments.

Environment	Principal component	QTL	Chromosome	QTL Position (cM)^a^	Flanking markers^b^	Peak LOD Score	Additive effect^c^	Gene Action^d^	R^2^ ^e^
**Coimbra**	PC1	pc1_c1	6	49.68	umc1186 / E36-M50-0151	4.35	-0.41	PD	13.4
		pc1_c2	8	52.64	E41-M60-0162	4.22	0.34	PD	13.1
	PC2	pc2_c1	7	93.41	E41-M60-0289 / E35-M47-0052	4.33	-0.13	OD	15.0
		pc2_c2	3	35.02	E36-M50-0127 / umc1449	4.34	0.21	PD	12.8
	PC3	pc3_c1	1	15.36	E32-M49-0277 / E35-M50-0215	6.37	-0.22	A	18.9
		pc3_c2	3	0.00	umc1057	3.89	-0.18	A	11.0
**Montemor**	PC1	pc1_m1	3	78.47	E32-M49-0720 / E35-M49-0385	7.63	-0.56	PD	22.4
		pc1_m2	1	79.11	E35-M18-0216 / E36-M62-0063	4.42	-0.23	OD	12.2
	PC2	pc2_m1	3	20.26	E41-M60-0475 / umc1392	5.43	0.22	PD	15.9
		pc2_m2	5	84.60	E32-M18-0195	4.31	-0.24	PD	12.4
		pc2_m3	7	91.41	E41-M60-0289 / E35-M47-0052	4.38	-0.14	OD	10.7
	PC3	pc3_m1	8	2.63	E35-M18-0169	4.43	-0.19	PD	15.3

^a^ QTL position in cM from the top of the chromosome

^b^ molecular markers flanking the support interval estimated at a LOD fall of -2.00

^c^ Additive effect = (phenotypic mean of the PB260 allele genotypes—phenotypic mean of the PB266 allele genotypes) / 2 [79]; negative values indicate that the PB266 allele increased trait additive value

^d^ Gene action: A—additive if |dominant effect/additive effect| < 0.2; PD—partial dominance 0.2 < |d/a| < 0.8, D—dominance 0.8 < |d/a| < 1.2; OD—overdominance |d/a| > 1.2

^e^ Percent explained phenotypic variance.

At Coimbra, two QTL were detected for PC1. The first in chromosome 6 (13.4% of the phenotypic variance explained), colocalized with QTL for medulla1 and 2, strongly correlated traits and highly contributing to this PC1 vector. The second in chromosome 8 (13.1% of explained phenotypic variance) colocalized with QTL for rachis traits, cob diameter 2 and cob weight, traits that were moderate to very strongly correlated and highly contributing for PC1 in this environment. Additionally, in Coimbra, two QTL were identified for PC2, in chromosomes 3 and 7. The chromosome 3 PC2 QTL (12.8% of explained phenotypic variance) colocalized with QTL for length, cob weight, medulla, and cob diameters, with some of these traits strongly correlated (ear length with cob weight, cob diameter 3 with 4 and medulla 1 with 2). In chromosome 7, PC2 QTL (15.0% of explained phenotypic variance) colocalized with a QTL for ear fasciation. Finally, two QTL were detected in this environment for PC3 in chromosome 1 (18.95% phenotypic variance explained), colocalizing with QTL for cob/ear weight per ear and kernel depth, traits that were weakly correlated, and in chromosome 3 (11.0% phenotypic variance explained), colocalizing with a kernel depth QTL detected on the same environment (Fig 1, Table 5).

At the Montemor environment, two QTL were identified for PC1. One QTL was located in chromosome 1 (12.2% phenotypic variance explained), colocalizing with QTL for row numbers, ear and cob diameter 1 (with row number 1 and 2 very strongly correlated), and another QTL in chromosome 3 (22.4% of the phenotypic variance explained), colocalizing with QTL for medulla and rachis traits, cob and ear diameters, with some of these traits very strongly correlated (ear diameter 1 and 2, ear diameter 3 and 4, cob diameter 1 and rachis 1, cob diameter 2 and rachis 2) (Fig 1, Table 5). Three PC2 QTL were detected for this environment in chromosomes 3, 5 and 7. In chromosome 3 (15.9% phenotypic variance explained), PC2 QTL colocalized with QTL for length, in chromosome 5 (12.4% of the phenotypic variance explained), with QTL for cob/ear weight per ear, cob weight and ear length, with some of these traits strongly correlated (cob weight with length and with cob/ear weight per ear), and in chromosome 7 (10.7% phenotypic variance explained), with QTL for all the ear diameters, cob diameter 3 and 4 and ear fasciation, with some of these traits strongly correlated (ear diameters 1 and 2, ear diameters 3 and 4, and fasciation with ear diameter 3). In the case of PC3, only one QTL was identified in chromosome 8 (explaining 15.3% of the phenotypic variance) and localized away from any clustering QTL region (Fig 1, Table 5).

As expected, and as already highlighted for the PC QTL, many of the highly correlated individual traits presented colocalized QTL. Overall, based on QTL two LOD confidence intervals, all the QTL detected for the 23 individual measured traits were summarized as 17 different QTL clustered regions (Fig 1), seven of which had in common three or more traits. Within these seven highly clustered regions, three presented constitutive QTL. In particular, colocalization of constitutive QTL was observed in chromosome 3, among QTL for all the medulla traits and for cob diameters 3 and 4, with strong correlations detected in both environments between cob diameters 3 and 4 and between medulla 1 and 2. In chromosome 5, colocalization was detected among QTL for cob weight, ear length and cob/ear weight per ear. Strong correlations existed among these traits, except for length and cob/ear weight per ear for both locations. Finally, in chromosome 8, colocalization was detected between QTL for cob diameter 1 and medulla 1 and strong correlations existed between these two traits in both studied environments (Fig 1, (S1 Table)).

### Putative candidate genes underlying detected QTL

From the 17 QTL regions, defined based on the QTL 2-LOD confidence intervals, we have selected eight different QTL regions to search for candidate genes (5 with constitutive QTL, plus 3 other with fasciation QTL or with QTL of fasciation highly correlated traits, ear and cob diameter 3 and row number 2) (Fig 2). On average, each region corresponded to 20.4 cM. Despite the exact physical distance covered by these intervals being unknown, several candidate genes, mapping to the defined QTL regions confidence intervals, have been identified from the literature, based on their potential biological function (Table 6).

**Table 6:** Candidate genes and previously described QTL for the currently detected ear fasciation and highly related traits QTL and other traits constitutive QTL.

	BIN	1.04–1.07	3.02–3.04	3.05–3.09	5.04–5.06	7.02-end	8.03–8.05										
	Marker interval	umc1144-umc1128	umc1057-umc1392	umc1907-umc1813	umc1221-umc1524	umc1585-end	phi125-umc1777										
	Detected QTL	ed3_c1 r2_m2 cd3_c1	l_c1 & m1	ed3_m2 cd3_c2 & m1 cd4_c1 & m1 m1_c3 & m3 m2_c3 & m1 r2_m1	l_c2 & m2 cw_c1 & m1 e_cwew_c2 & m1	ed3_m1 fa_c1 & m1 cd3_m2	cd1_c1 & m2 m1_c1 & m1	Fasciation	Meristems	Ear	Tassel	Flowering	Tillering	Interaction	Homologue	Homology/Protein	References
**Candidate Gene/QTL**	*barren inflorescence2 (bif2)*	1.05								x	x		x	*ba1*		/serine threonine protein kinase	[11,26–29,31,41]
	QTL cob diameter 6	1.07								x							[32,33]
	QTL kernel row 23	1.07								x							[10,35]
	QTL cob diameter 12	1.07								x							[34]
	QTL cob diameter 24	1.07								x							[10,35]
	QTL cob diameter 28	1.07								x							[10,35]
	*ramosa2 (ra2)*		3.02–3.03					x	x	x	x			*ra1*	*ra1*, *ra3*	LOB-domain TF/	[37,38,41,44,77,80–82]
	*tasselseed4 (ts4)*			3.04–3.05					x	x	x			*ids*, *ts6*, *sid1*		miR172/miR172 microRNA	[40–42,77,83–85]
	*terminal ear1 (te1)*			3.05				x		x	x				*ra1*, *ra3*	RNA-binding/RNA binding protein	[41,56,77,86–89]
	*bearded-ear1 (bde1)*				5.06				x	x		x					[45,50,77,90]
	*ramosa3 (ra3)*					7.04		x	x	x				*fea1*	*ra1*	/Trehalose-6-phosphate phosphatase	[38,41,57,77,81,91–93]
	*branched silkless1 (bd1)*					7.04–7.06				x	x					AP2-domain TF	[41,47,48,77]
	*compact plant1 (ct1)*						8.01–8.03	x		x							[51,94–97]

Main gene interactions and known homologies.

In the chromosome 1 region flanked by umc1144 and umc1128, where QTL for rows number 1 and 2, ear and cob diameter 1 (Montemor) and ear and cob diameter 3 (Coimbra) were colocalized, a possible candidate gene was *barren inflorescence 2* (*bif2*).The *bif2* is associated with maize architectural diversity and is known to affect the transition from inflorescence meristem to spikelet pair meristem or branch meristem [11, 26–28]. The *bif2* mutants have defects in the initiation of axillary meristems, and consequently, produce a reduced number of tassel branches and spikelets [26]. Additionally, *bif2* mutants also produce a reduced number of ears, with fewer kernels, and their apical meristem is often fasciated [29]. The *bif2* gene encodes a serine/threonine protein kinase that regulates polar transport of auxin [27]. BIF2 interacts with and phosphorylates BARREN STALK1 (BA1), a basic helix–loop–helix (bHLH) transcription factor required for axillary meristem initiation, suggesting that BA1 is a target of BIF2 [29–31].

In this same region, several QTL for cob diameter [32–34] and kernel row number 23 [10,35] were also previously detected (Table 6).

In the chromosome 3 region, ranging from umc1057 to umc1392, where the constitutive QTL for ear length was identified, we found *ra2* as a potential candidate gene. The *ra2*, similarly to *ra1*, has a highly branched and distorted ear, with irregular kernel placement [36]. The *ra2* gene encodes a LOB [37] domain protein that determines the fate of stem cells in maize branch meristems [37,38]. The *ra2* regulates accumulation of *ra1* transcripts, placing the two genes in a single genetic pathway, with *ra2* upstream of *ra1* [36].

In the chromosome 3 region, ranging from umc1907 to umc1813, where the constitutive QTL for cob diameters 3 and 4 and medulla 1 and 2, plus ear and cob diameters, rachis and rows number for Montemor were identified, we found as a candidate gene the *tasselseed4* (*ts4*). The *ts4* encodes a mir172 microRNA that controls sex determination and meristem cell fate by targeting *Ts6*/*indeterminate spikelet1* (*ids1*) [39]. In addition, *ts4* not only affects sex determination, but can also cause inflorescence branching proliferation due to acquired indeterminacy of the spikelet pair meristem and the spikelet meristem [39–41]. The *ts4* mutants are characterized by irregular branching within the inflorescence and feminization of the tassel caused by a lack of pistil abortion [42].

Also in the same region of chromosome 3 (range umc1907 to umc1813), we found an additional potential candidate gene, *terminal ear1* (*te1*). Mutants of the maize *te1* gene have shortened internodes, abnormal phyllotaxy, leaf pattern defects and partial feminization of tassels. An earlike inflorescence forms in place of the normal terminal tassel. There is an increase in the frequency of leaf primordia initiation and the feminization of the terminal inflorescence on the main stalk [43,44]. The *te1* gene encodes a RNA recognition motif (RRM) protein, and is expressed in the vegetative shoot apex, in semicircular rings, that laterally oppose the positions of leaf primordia [43].

In the chromosome 5 region, ranging from umc1221 to umc1524, where the constitutive QTL for cob weight, cob/ear weight per ear and ear length plus the QTL for ear weight, were detected only in Coimbra, the *bearded-ear1* (*bde1*) was indicated as a potential candidate gene. The *bde1* encodes *zea agamous3* (*zag3*), a MADS box transcription factor belonging to the conserved AGAMOUS-LIKE6 clade [45]. The *bde1* is critical for multiple aspects of floral development, including floral meristem determinacy, organ development and sex determination. The *bde1* mutation affects floral development differently in the upper and lower meristem [45]. The upper floral meristem initiates extra floral organs that are often mosaic or fused, while the lower floral meristem initiates additional floral meristems.

In the chromosome 7 region, ranging from umc1585 to the end of the chromosome, where QTL for fasciation, cob diameter 3 and related traits were identified, we have detected as a potential candidate gene *branched silkless1* (*bd1*)[46], which encodes an ERF-like APETALA2 transcription factor and functions to repress indeterminate lateral branch meristem fates [46]. The *bd1* was first described by Kempton [47]. In mutants with strong alleles the ear spikelet meristems are replaced by branches similar to the tassel. In addition, no florets are initiated in the ear. In the tassel, the phenotype is less severe, possibly due to the expression of the duplicate of *bd1*. While the tassel spikelets are still indeterminate and branch-like, florets are initiated that produce viable pollen. The *bd1* is expressed in boundary domains, adjacent to the meristems that *bd1* also regulates. Phylogenetic analysis of the *bd1* gene demonstrated high conservation of the gene in all grass lineages with spikelets, indicating that the gene may be fundamental to spikelet initiation [48,49].

In the same QTL region we also found the *ra3* as a potential candidate gene. In its mutants, the axillary meristems can be enlarged and acquire abnormal identity or became indeterminate, leading to the production of long branches or more floral meristems in the ears. Tassels present the same developmental defects, although at a lower frequency, leading to additional long branches [49]. The *ra3* encodes a functional trehalose-6-phosphate phosphatase, an enzyme that catalyzes the production of trehalose sugar and is expressed in discrete domains subtending axillary inflorescence meristems [50]. Genetic analysis has placed all three *ramosa* genes into a pathway, with *ra2* and *ra3* acting in parallel upstream of *ra1* [50]. It was proposed that RA3 regulates inflorescence branching by modification of a sugar signal that moves into axillary meristems. Alternatively, the fact that RA3 acts upstream of RA1 supports the hypothesis that RA3 itself may have a transcriptional regulatory function [50].

Finally, in the chromosome 8 region, ranging from phi125 to umc1777, where the constitutive QTL for cob diameter 1 and medulla1 were located, we have identified as a potential candidate gene the *ct1*, whose mutant phenotype depicts semidwarf plants with furcated ears, but not fasciated, with all plant parts reduced proportionately [51].

## Discussion

Fasciation is frequently found in the Portuguese maize germplasm [22]. The knowledge of its genetic control could be used to better modulate yield while controlling the negative secondary effects of extreme fasciation expression (e.g. increasing yield, but maintaining uniformity of plants and ears) [25]. However, molecular genetic studies to understand the genetic basis of this trait were never performed on Portuguese germplasm.

To determine the genetic relationships among a comprehensive set of maize ear architecture traits related with fasciation, the current study presents a QTL analysis of the ear fasciation and related traits, for the first time undertaken on maize germplasm of Portuguese origin. This study also allowed us to propose potential candidate genes for ear fasciation. The results were obtained by repeated phenotypic analysis of the ear fasciation and related traits using a segregating F_2_ maize population of Portuguese origin (non-fasciated PB260 x fasciated PB266) that was also genotyped with AFLP and SSR markers.

QTL analysis revealed the existence of non-constitutive QTL for fasciation indicating a possible contribution of some minor environment-specific genes. However, also a limited experimental significance could be the cause for this non-detection considering our experimental limitations and the reduced number of environments tested. Even though polygenic [1,6,25], the inheritance of the ear fasciation trait in the germplasm of Portuguese origin was not particularly complex (four QTL were detected for ear fasciation, one of them constitutive in the two studied environments), paving the way for relatively straightforward use of molecular markers in breeding programs by exploiting ear fasciation control.

In addition, 10 QTL were detected for the highest fasciation correlated traits (3 for ear diameter 3 and row number 2, and 4 in the case of cob diameter 3).

The number of detected QTL may have been underestimated, as QTL, may have escaped detection due to the scarcity of markers in some map regions, the small F_2_ population size (starting from 149 individuals) and a relatively high LOD threshold score used to reduce the rate of false positives [52]. Future work should focus on the saturation of the genetic map presented here, with more codominant markers or other types of higher throughput dominant markers (such as Single Nucleotide Polymorphisms, SNP). This would allow gaps between distant markers to be filled, as well as increasing the likelihood of merging the total amount of screened markers into 10 linkage groups.

Even so, the presently detected QTL, considering the absence of epistasis and not assessing the variation potentially explained by QTL interactions could explain, per environment (Coimbra and Montemor), 24.7% and 26.4% of the phenotypic variation for ear fasciation; 14.3% and 30.8% of the phenotypic variation of ear diameter 3; 27.6% and 34.7% of the phenotypic variation of cob diameter 3; and 43.1% of the phenotypic variation of row number 2 (although in this last case, these QTL were only detected at Montemor).

In the F_2_ population, a high correlation between ear fasciation and ear and cob diameter 3 and row number 2 was observed. This observation was consistent with the fact that the QTL for ear fasciation were colocalized, depending on the environment, at least with QTL for ear diameters 1 to 4 and cob diameters 3 to 4. QTL for ear fasciation and row number 2 were detected on the same chromosome (chromosome 2) but there was no overlapping of the respective confidence intervals. Nevertheless, taking into account the small population size, the non-overlapping of the two-LOD intervals of the particular ear fasciation and row number 2 QTL in chromosome 2 might have been caused by the choice of particular cofactor markers during the multiple QTL mapping approach.

The parental accession PB266 had an average higher level of ear fasciation than the parental accession PB260. Still, the alleles contributed by the parental accession PB266, in all the detected ear fasciation QTL including the constitutive QTL, decreased trait additive value. A similar situation occurred for the related trait row number 2, cob diameter 4, ear diameter 3 and 4 and cob/ear weight per ear detected QTL where, although the parental accession PB266 had significantly higher phenotypic values than the parental accession PB260, it contributed not only with alleles increasing, but also with alleles decreasing trait additive values. In fact, transgressive segregation was observed for the majority of the analyzed traits, with phenotypic values of the two parental accessions significantly different from at least one of the two extremes of the F_2_ individuals’ phenotypic range.

The knowledge of the genetic basis and location of the QTL responsible for the ear fasciation expression as well as its potential interaction with other related traits will facilitate the transfer of the milder fasciation alleles from the Portuguese germplasm to modern cultivars, hopefully without the negative effects of an extreme fasciation expression. As already highlighted, molecular markers could be used to support this introgression. In order to achieve this goal, the search for candidate genes as functional markers, arguably more promising and efficiently than the flanking markers for selection, is extremely important, providing that a direct association between function and phenotype exists.

The only constitutively detected fasciation QTL was located in chromosome 7, overlapping with QTL for ear and cob diameters, these last ones only detected in Montemor. Some of the possible candidate genes for fasciation in this position are *bd1* and *ra3* (mapped in the bin 7.04), which indeed can be related with the QTL for ear and cob diameters and fasciation identified in this chromosome 7 region (Table 6). The *bd1* affects ear branching architecture, being fundamental to spikelet initiation, and so could influence the ear fasciation or diameter traits presently studied. The *ra3* is of great value for ear architecture and establishes the correct identity and determinacy of axillary meristems in both male and female inflorescences. However, previous studies with Portuguese maize fasciated germplasm, which did not consider the inbred lines that gave rise to the presently studied population, showed that the abnormal ear expression was not allelic to the three *ramosa* genes [25]. Yet in the present study *ra3* is seen as an interesting candidate gene. This support the existence of diversity in the genetic control of the fasciation expression among Portuguese maize germplasm, *i*.*e*., different Portuguese germplasm may contain different combinations of different genes, all resulting in ear fasciation. In addition, the *ra2* was identified in the present study as a potential candidate gene, not for fasciation, but for ear length, in chromosome 3. Indeed, Pêgo and Hallauer studies [25] stated that the genetic potential for increased yield in the fasciated Portuguese germplasm would be conditioned by the interaction between fasciation expression and ear length. In the present study, no strong negative correlation was detected between fasciation and length of the ear (-0.322 and -0,141 respectively for Coimbra and Montemor) (S1 Table), probably due to the Portuguese farmers’ selection criteria, which preferred long ears (although with fasciation expression). But these two traits are known to vary in opposite directions [5]. In the present study we observed that the two parental accessions behave oppositely. PB260 was contributing positively to fasciation increase and negatively to ear length, while PB266 was contributing negatively to fasciation and positively to ear length increase in the studied population (Table 2).

In the present study, other fasciation QTL were detected in chromosome 2 and 10, but not constitutively, indicating that these are also chromosomal regions that can be further explored to fully understand the genetic control of ear fasciation in the Portuguese germplasm. A QTL for row number 2 (only for Montemor) was also detected in this study in the chromosome 2 region, but in a different region from the fasciation QTL. In chromosome 10 QTL have been previously detected for grain yield (bin 10.03) [53,54] and kernel row number per ear (bin 10.03–10.07) [55]. The ear fasciation trait had, in our study, a low correlation with yield; however, correlation with row numbers 1 and 2 (0.63 and 0.75, respectively) (S1 Table) were much higher. Nevertheless, perhaps due to the lower resolution power of the present study, no constitutive kernel row number QTL was detected, and overall only for the Montemor environment did we detect kernel row number QTL in chromosomes 1, 2 and 3.

Also in chromosome 3, but away from the *ra2* location, QTL for ear diameter 3, row number 2 and cob diameter 3 (this one constitutively localized), were detected together with constitutive QTL for cob diameter 4 and medulla 1 and 2. Some of these traits had the highest correlation coefficients with fasciation (ear and cob diameters 3 and row number 2). In this region *ts4* and *te1* could be considered as potential candidate genes for the aforementioned fasciation-related traits, due to their associated increased ear branching phenotypes, similar to a certain extent to what is found in the typical Portuguese maize fasciated traditional varieties. The *ts4* mutant phenotype presents a tassel compact silky mass, upright, with pistillate and staminate florets, with a proliferated, silky ear [40,41]. In the *te1* mutant, kernel rows may be uneven and branches may form in the ear, depending on the allele and background [56].

Another constitutively detected QTL for ear length found in this study was located in chromosome 5, overlapping with the constitutively detected QTL for cob weight and cob/ear weight per ear. These traits were highly correlated with coefficients ranging from 0.69 to 0.98 (S1 Table). A candidate gene in this chromosomal region might be the *bde1* (bin 5.06). Its mutants present polytypic and silky ears, showing a proliferation of pistillate tissue causing irregular growth on the ear and tassel. These phenotypes may indicate a possible influence of this gene on the traits for which QTL were detected in this position. Furthermore, in this same chromosome 5, the *fea1* [57] could also be indicated as an important candidate gene for these detected QTL, due to the small rounded ears and fasciated inflorescence meristems associated mutant phenotype, but its precise location is not yet known [57].

In a chromosome 8 region (8.03–8.05), we constitutively detected two QTL for cob diameter 1 and medulla 1, traits that are strongly correlated. A possible candidate gene in this interval is the *ct1*, whose mutant phenotype depicts semidwarf plants with furcated ears, but not fasciated, with all plant parts reduced proportionately [51]. Furcated ears are very often observed among the Portuguese fasciated germplasm, with a strong effect on cob and medulla ear traits.

Other candidate genes related to ear fasciation, such as the *bif2*, were previously located in chromosome 1 [26], within the interval where we detected QTL for traits such as ear and cob diameter and row numbers, some of them highly correlated with ear fasciation (ear and cob diameter 3 and row number 2). The *bif2* mutants produce a reduced number of ears with fewer kernels. In addition, the apical meristem is often fasciated [26,31], a trait that is highly correlated with ear and cob diameters and row numbers. In this same region several ear traits QTL have also been previously detected by others. This is the case of the QTL cob diameter 6 (in bin 1.07) [32,33] and QTL kernel row number 23 [10]. Indeed, this chromosomal region appears to be highly associated with the inheritance of cob diameters, since other cob diameter QTL were also mentioned as near some of those internal markers (in bin 1.07), such as QTL for cob diameter 12 [34], 24 and 28 [10].

In an attempt to identify the genomic regions controlling the most important factors contributing to the definition of the overall variation in maize ear architecture and yield, we detected QTL for the Principal Components calculated separately for each environment. Colocalization of PC QTL and individual traits QTL was in accordance with the main contribution of each individual trait for each PC. Accordingly, in Coimbra, PC1 QTL overlapped individual QTL for cob weight, cob diameter, medulla and rachis in chromosomes 6 and 8; PC2 QTL overlapped individual QTL for ear length in chromosome 3 and, interestingly, also overlapped the constitutive ear fasciation QTL in chromosome 7, although fasciation is not one of the most contributing traits for this component. PC3 QTL overlapped individual QTL for kernel depth and cob/ear weight per ear in chromosome 1 and 3 (S2 Table). In Montemor, PC1 QTL colocalized with individual QTL for cob and ear diameters in chromosome 1 and QTL for rachis, medulla and ear and cob diameters in chromosome 3. Also in chromosome 3, PC2 QTL overlapped with ear length QTL, and the same happened in chromosome 5. As already pointed out, Coimbra PC2 QTL also overlapped with the constitutive fasciation QTL in chromosome 7. Finally, the QTL detected for PC3 in Montemor did not overlap with any of the individual trait QTL in chromosome 8 (S2 Table). Indeed for this principal component no individual trait contributed in a outstanding way. Possibly this QTL might be involved in a more overall regulation of multiple ear traits, which could not be detected using trait-by-trait analysis [11]. This fact reinforces the existence of recently detected regions that can be further explored in order to find new associations between QTL traits and candidate genes and to better understand and control fasciation in maize breeding.

Pêgo and Hallauer [25] concluded that ear fasciation is a complex trait important in the Portuguese maize germplasm, with high potential for long-term maize breeding. In our study, since we used a segregating population developed from crossing only two contrasting inbreeds, we might have missed many of the alleles that control this ear trait in the Portuguese germplasm. In order to clarify which other genes or alleles are contributing to the fasciation expression in this interesting maize germplasm, future mapping approaches should consider multiparental populations or association mapping with an higher number of Portuguese-derived inbreed lines such as the ones described in Vaz Patto et al. [58].

In relation to the currently proposed candidate genes, fine mapping with additional markers in advanced Recombinant Inbred Lines (RIL) populations or complementary testing using Near Isogenic Lines (NIL) will be needed for the validation of some of the present hypothesis.

The present work represents the first molecular study in the elucidation of a set of genes controlling fasciation and associated molecular markers from the long-term legacy of Portuguese farmers, which after validation might have important breeding applications.

Portuguese farmers’ selection of maize occurred over centuries and counted on the fasciation ear trait to increase ear size and yield. However, high levels of ear fasciation are associated with abnormal ear shapes that seriously limit harvesting. Additionally, its quantitative expression due to its genetic complexity and dependency on environmental conditions hinders its current application in breeding programs. This is a very interesting trait for maize breeding, but one that must be fully understood at a genetic level before perfectly controlled in breeding programs. This control can be attained by the development of molecular selection tools based on QTL flanking molecular markers or associated functional markers (candidate genes), such as the ones identified in the present study. This study represents the first steps into the development of biotechnological tools for Marker Assisted Selection (MAS) of ear traits related to the typical fasciation of Portuguese maize germplasm. However prior to this, the newly identified QTL regions should be saturated with more molecular markers to increase the precision of QTL location and the linked flanking markers should be validated in other breeding populations. In our particular case a collection of diverse Portuguese maize inbred lines or the ear fasciation contrasting traditional maize landraces could be used to test if these trait /marker associations would be maintained in other genetic backgrounds. This breeding approach would ensure the use of a proper combination of genetic factors controlling ear diameter, kernel row number and ear length to allow ear fasciation expression without abnormal ear shapes and increasing yield and/or ear size, depending on the final breeding objective [59].

## Conclusions

We have detected significant variation for maize ear fasciation and related ear traits and mapped a number of QTL controlling those traits in the Portuguese derived PB260 x PB266 segregating population. We have found a substantial positive genetic correlation between ear fasciation and ear diameter 3, row number 2 and cob diameter 3, with heritabilities higher than 0.73. The constitutively detected QTL for fasciation was located in chromosome 7, indicating *ra3* as a putative candidate gene. This QTL mapping study has contributed to expanding the list of genomic areas involved in maize ear fasciation and related traits, especially in chromosomes 1, 3, 5, 7 and 8 where candidate genes *bif2*, *ra2*, *ts4*, *te1*, *bde1*, *ra3*, *bd1* and *ct1* and associated molecular markers were proposed.

## Materials and Methods

### Population development

Based on information from the records of NUMI (a national maize breeding station in Braga, Portugal), two contrasting inbred lines for ear fasciation, PB260 (non-fasciated) and PB266 (fasciated) were selected as parental lines for the development of a fasciation segregating population. NUMI targets were the Portuguese farmers, who mainly used maize for bread production, *i*.*e*., they selected mainly for white flint kernels with white cobs.

The PB260 pedigree is (PB6 x PB7) x PB6(2), PB6 being an inbred line derived from the Portuguese landrace ‘Cem dias’ and PB7 an inbred line derived from ‘Northern White’, an American population. PB260 was selfed for 19 years. From three years of field evaluations, PB260 presented an average of 72 days for male and female flowering, and in a scale from 1 to 4 (where 1 is the minimum and 4 the maximum), 1.3 for vigor, 2 for plant height, 4 for uniformity of the plants in the plot, 2 for plant lodging and 1.2 for *Sesamia* spp. resistance. The ear height insertion was 3, in a scale from 1 to 9 (where 1 is the minimum and 9 the maximum and 5 corresponds to the middle of the plant). The ear shape was conical, with white flint kernel type and white cob, with a fasciation level of 1.41 in a scale from 1 to 9 (where 1 is the minimum and 9 the maximum).

The pedigree of PB266, also known at NUMI as WF9R, is (WF9 x PB53) x WF9. The WF9 is a yellow dent inbred line originally selected by the Indiana Agriculture Experimental Station from the population Wilson Farm Reid [60]. Historically, the name of WF9 was kept at NUMI, although when introduced into the Portuguese breeding program in the 40s, this yellow dent kernel line, as many others, was converted to a white inbreed line by crossing with Portuguese germplasm. In particular, this conversion included crosses with Portuguese germplasm with white abnormal ears, followed by several backcrosses to the recurrent parents [25]. The PB53 was derived from ‘Northern White’, an American population [61]

The PB266 line was also selfed for 19 years, before being used in this study. PB266 is characterized by 74 days for male and female flowering, and in the same 1 to 4 scale described before, it presents 1.2 for vigor, 3 for plant height, 4 for uniformity of the plants in the plot, 3 for plant lodging and 3.5 for *Sesamia* spp resistance. Following the 1 to 9 scale, its ear height insertion was also 3. The ear shape was conical, with white dent kernel type and white cob, with a fasciation level of 2.38. Due to the relatively small ear fasciation differences among PB260 and PB266, this cross allowed us to identify genes contributing to a milder ear fasciation expression variation.

Vaz Patto et al. [58] studying the genetic diversity of a collection of Portuguese maize inbred lines, clustered PB260 together with white flints of a Portuguese origin. PB266 was not analyzed in that study however, its genetic distance, later computed, to WF9 was 0.197, while to PB260 it was 1.063 (Alves ML, unpublished results), which indicates PB266 clustering nearby WF9, on the yellow dent germplasm group of American origin, and away from PB260. Indeed PB266 was selected to be the Portuguese WF9 version, *i*.*e*., with white kernel and cob, and with an early cycle more adapted to the national farming systems and more resistant to *Sesamia* spp.

PB260 and PB266 were crossed to develop an F_1_ hybrid. A F_1_ hybrid plant was self-pollinated to obtain an F_2_ population. 149 randomly chosen F_2_ plants were selfed to obtain 149 F_2:3_ families. Leaf samples were collected from each of the 149 F_2_ plants for DNA extraction and molecular markers analysis. The F_2:3_ families derived from the 149 F_2_ individuals were used to evaluate ear fasciation and related ear architecture traits. The evaluation occurred under field and laboratory conditions.

### Field experiments and phenotypic evaluations

The 149 F_2:3_ families were evaluated at two environments in Portugal (Coimbra 40°13'0.22"N, 8°26'47.69"W and Montemor 40°10'4.82"N, 8°41'14.84"W) in 2008. These two environments are a part of the Mondego irrigation perimeter, a very high-yielding area where the average yield for maize hybrids is 14.5 Mgha^-1^. Montemor is located 21 km from the sea coast and Coimbra 50 km. Both environments have an altitude of 25m. Both Montemor and Coimbra have alluvial soils, but compared to Montemor, Coimbra has a lower soil pH (5.2 versus 6.3) and a lower percentage of soil with a particle size less than 0.2 mm diameter (86.9% versus 92.5%); it also has a higher percentage of organic matter (2.3% versus 1.7%). The agricultural practices were similar in both environments; however the sowing date in 2008 was May 9 at Coimbra and May 28 at Montemor and the harvest from October 2 and 21, respectively.

In each environment, a randomized complete block design, with two replications, was used. Each plot consisted of one single row with 3.1 m (2.6 m planted row plus 0.5 m, space between two planted rows) long, with an inter-row distance of 0.75 m. Each plot was overplanted by hand and thinned at the V7 growth developmental stage [62] for a stand of approximately 50000 plants ha^-1^. Plots were mechanically and/or hand-weeded and managed following common agricultural practices for maize in the region. All the plots were harvested by hand.

Phenotypic data were collected for 29 traits and are described in Table 1. Some traits were measured per plot (traits 1–4, Table 1), such as grain yield (Mgha^-1^) adjusted to 15% grain moisture at harvest. All the other traits were measured on five ears per plot, randomly selected after harvest, and dried (35°C) to approximately 15% grain moisture to ensure *ceteribus paribus* conditions during measurements. Following this procedure, 25 measurements were made per ear (traits 5–29, Table 1) [23]. The five ears average value per plot was considered for data analysis (Table 1).

### Statistical analysis of phenotypic data

Phenotypic data descriptive statistics were calculated using SAS (the SAS system for Windows, version 9.2, Cary, NC, USA). Pearson's correlation coefficients were computed for each trait between environments as well as between all traits by PROC CORR procedure. The distributions of the traits in each environment were tested using the Kolmogorov-Smirnov's test of normality. PROC GLM procedure was used for analysis of variance. Environments (Coimbra and Montemor) and genotypes were treated as fixed effects. Repetitions, treated as random, were nested in the environments. Genotype x Environment interaction was included in the model. The PROC VARCOMP was used to estimate variance components for each trait in each environment separately as well as for both environments. Broad-sense heritabilities, representing the part of the phenotypic variance in the total phenotypic variance, were calculated for each environment as: h^2^ = V_g_^2^/ [V_g_^2^ + (V^2^/r)], where V_g_^2^ is the genotypic variance, V^2^ is the error variance and *r* is the number of replications, and for both environments as: h^2^ = V_g_^2^/ [V_g_^2^ + (V_ge_^2^/e) + (V^2^/re)], where V_ge_^2^ is the G x E interaction variance and *e* is the number of environments.

In order to have an indication of possible transgressive segregation among parental lines and the F_2:3_ families, we compared the average data of PB260 and PB266 obtained at Coimbra during 2010 and 2012 field trials, with two repetitions in organic production, with the average extremes of the F_2:3_ families field trials (obtained as described in field experiments and phenotypic evaluations section). For the extremes of the F_2:3_ families we considered the top five maximum and top five minimum values per trait. Analysis of variance was applied to these data. When significant differences were detected, the Shéffe test was used to compare parental and extreme F_2_ averages (Table 2).

Principal component analysis (PCA) was performed using PROC PRINCOMP procedure in SAS considering all phenotypic traits separately for each environment in order to isolate the most important factors contributing to the definition of the overall variation in maize ear architecture and yield. The first three principal components were used to map QTL associated with the overall variation of maize ear architecture and yield in a multivariate approach.

### Marker analysis and linkage map construction

Plant leaf samples were collected from the 149 F_2_ PB260xPB266 individuals at V9 to V12 stages of growth and development, from the self-pollination field trial. These 149 F_2_ self-pollinated individuals produced sufficient seed for establishing the F_2:3_ families’ multilocation trials.

DNA was extracted from each F_2_ plant leaf sample using a modified CTAB procedure [63]. The F_2_ population individuals were analyzed using Simple Sequence Repeats (SSRs) and Amplified Fragment Length Polymorphism (AFLP) markers.

#### SSR protocol

The SSR marker technique was performed as described by Vaz Patto et al. [58] with minor modifications. Forward primers of SSR primer pairs were labeled with two fluorescence dyes (IRDye 700 or IRDye 800) (Eurofins MWG Operon, Germany) to allow amplification fragments analysis using an 4300 DNA analyzer system (LI-COR Biosciences, USA). SSR alleles were detected and scored using SAGA Generation 2 software (LI-COR Biosciences, USA).

In order to select the most informative SSR primer pairs, the parental lines, PB260 and PB266, and a F_1_ individual were screened with 211 SSR markers chosen from Maize Genetics and Genomic Database (MaizeGDB) [64] based on their repeat unit and bin location. This resulted in the selection of 60 SSR primer pairs that were amplified on the F_2_ individuals. Primer sequences are available from the MaizeGDB.

The amplification fragments size was determined in base pairs and visually scored (peak detection) at least twice independently for each entry, to ensure data accuracy. Data were recorded as present (1), absent (0) or missing (-), allowing the construction of a binary matrix of the SSRs phenotypes.

#### AFLP Protocol

The AFLP technique was performed using the AFLP Analysis System I (Invitrogen, Carlsbad, CA, USA) kit protocol, with minor modifications. The *EcoRI* primers were labeled with two fluorescence dyes (IRDye 700 or IRDye 800) (Eurofins MWG Operon, Germany) to allow amplification fragments analysis using an 4300 DNA analyzer system (LI-COR Biosciences, USA). *MseI* primers with only two selective nucleotides were also tested to increase the total number of amplified fragments per primer combination. The primer core sequences were those of Vos et al. [65]. Thirty-six *Eco*RI/*Mse*I base primer combinations were first tested in the parental lines (PB260 and PB266) in order to select the most informative primer combinations. This resulted in the selection of 17 different primer combinations that were used to screen the 149 F_2_ individuals.

Clearly readable amplified fragments of the 149 accessions were determined for size in base pairs and visually scored at least twice independently for each entry; they were recorded as present (1), absent (0) or missing (-) [60].

This allowed the construction of the binary matrix of the different AFLP phenotypes. This matrix, together with the SSR data matrix, was used for the construction of the input file for JoinMap 4.0 software [66].

#### Map construction

Linkage analysis and segregation distortion tests (P≤ 0.05) were performed using JoinMap 4.0 software [66]. The determination of linkage groups of markers was done with a LOD score of 3. The linkage map calculations were done using all pairwise recombination estimates lower than 0.49 and a LOD score higher than 0.01, and applying the Kosambi mapping function [67].

Individuals and markers with more than 10% missing values were removed from the original molecular data set. Also markers with a severe segregation distortion (P≤ 0.005) were excluded.

After a preliminary map analysis, improbable genotypes, including double recombination events (singletons), markers with suspected linkages with other markers and redundant markers clustered at the same position were removed, following the approach of Vaz Patto et al. [68]. All of the codominant markers were kept in this refined map. To check the reliability of the obtained map, the individual linkage group χ^2^ was inspected.

Linkage groups were assigned to the corresponding chromosome using the SSR map locations from the consensus maize map as in the Maize Genetics and Genomics Database, MaizeGDB [49]. This was also a check for the accuracy of the composition of linkage groups, as only markers assigned to the same chromosome should be present in the same linkage group.

### QTL analysis

The previously obtained F_2_ refined linkage map was used for QTL identification. Kruskal-Wallis single-marker analysis (non-parametric test), as well as for both interval mapping [69] and multiple-QTL mapping (MQM) [70] were performed using MapQTL version 4.0 [71]. A backward elimination procedure was applied to select cofactors significantly associated with each trait at P < 0.02 to be used in MQM. Genome-wide threshold values (P < 0.05) for declaring the presence of QTL were estimated from 10,000 permutations of each phenotypic trait [72]. The 1-LOD and 2-LOD support intervals were determined for each LOD peak.

The R^2^ value, representing the percentage of the phenotypic variance explained by the marker genotype at the QTL, was taken from the peak QTL position as estimated by MapQTL. Additive and dominance effects for detected QTL were estimated using the MQM procedure. Gene action was determined following Stuber et al. [73] as: additive (d/a = 0–0.20); partial dominance (d/a = 0.21–0.80); dominance (d/a = 0.81–1.20); and overdominance (d/a > 1.20), where, d/a = dominance effects/additive effects. Maps were drawn using MapChart version 2.2 software [74]. QTL analysis was performed on entry means from individual environments. The QTL nomenclature corresponded to the trait’s abbreviation (Table 1) followed by the environment abbreviation (c = Coimbra and m = Montemor), and finally a rank number, indicating the contribution of the QTL for a certain trait (based on R^2^) (Table 4).

QTL for different traits were declared as potential ‘‘common QTL” when they showed overlapping confidence intervals [75]. On the other hand, “constitutive QTL” referred to a stable QTL across both environments [76].

Potential candidate genes and previously published QTL were identified for the ear fasciation and highly related traits and for all the constitutive QTL regions. This search was performed by comparing the 2-LOD confidence interval positions of the presently detected QTL with the known locations of genes and QTL affecting yield and ear architecture traits at the consensus Maize IBM2 2008 Neighbours Frame Map, available from MaizeGDB [49]. The presently detected 2-LOD confidence interval SSR flanking markers were used as anchor markers in these map comparisons. The qTeller toolbox [77] was also helpful on the QTL position comparisons.

## Supporting Information

S1 Table Phenotypic correlations among ear fasciation and related traits with respective P-value for Coimbra (above the diagonal) and Montemor (below the diagonal). The diagonal represents, for the same trait, the correlations between locations. ^a^ squares diagonal indicates the Pearson coefficient correlation (r) for each trait between the two environments and respective P-value (very strong correlation (0.90 to 1.00) in black; strong (0.70 to 0.90) in dark grey and moderate (0.65 to 0.7) in light grey). Traits measured: yld—yield; cwew—cob/ear weight at harvest; en—ears number; av_ew—20 ears average weight at harvest; l—ear length; ed 1 to 4—ear diameter 1 to 4; cd1 to 4—cob diameter 1 to 3; kd—kernel dept; m1, m2—medulla 1 and 2; rq1, rq2-rachis 1 and 2; ew-ear weight; cw-cob weight; sw-thousand kernel weight; kw—kernel weight; e_cwew—cob/ear weight per ear; r1, r2—kernel-row number 1 and 2; fa—fasciation; cv—ear convulsion; kn—kernel number; nc—kernel per row
(DOCX)

S2 Table Component loadings for the first three Principal Components (PC) of 29 ear fasciation and related traits in the maize F_2_ (PB260xPB266) in two environments. ^a^ PC loading scales correlation in absolute values: very weak: 0.00 to 0.20; weak: 0.20 to 0.40; moderate: 0.40 to 0.70; strong (in grey): 0.70 to 0.90; very strong (in black): 0.90 to 1.00. Levels of significance: ns non-significant value; * significant at P < 0.05; ** significant at P < 0.01; *** significant at P < 0.0
(DOCX)
