# Supplementary material for: Genetic Architecture of Ear Fasciation in Maize (Zea mays) under QTL Scrutiny
Source: PLoS One. 2015 Apr 29;10(4):e0124543. doi: 10.1371/journal.pone.0124543 (PMC4414412; doi:10.1371/journal.pone.0124543)
Supplement: S1 Table — The diagonal represents, for the same trait, the correlations between locations. a squares diagonal indicates the Pearson coefficient correlation (r) for each trait between the two environments and respective P-value (very strong correlation (0.90 to 1.00) in black; strong (0.70 to 0.90) in dark grey and moderate (0.65 to 0.7) in light grey). Traits measured: yld—yield; cwew—cob/ear weight at harvest; en—ears number; av_ew—20 ears average weight at harvest; l—ear length; ed 1 to 4—ear diameter 1 to 4; cd1 to 4—cob diameter 1 to 3; kd—kernel dept; m1, m2—medulla 1 and 2; rq1, rq2-rachis 1 and 2; ew-ear weight; cw-cob weight; sw-thousand kernel weight; kw—kernel weight; e_cwew—cob/ear weight per ear; r1, r2—kernel-row number 1 and 2; fa—fasciation; cv—ear convulsion; kn—kernel number; nc—kernel per row (DOCX) [file pone.0124543.s001.docx]

**Table S1** Phenotypic correlations among ear fasciation and related traits with respective P-value for Coimbra (above the diagonal) and Montemor (below the diagonal). The diagonal represents, for the same trait, the correlations between locations

| **m\c** |  | **yld** | **cwew** | **l** | **de1** | **ed3** | **ed2** | **ed4** | **r1** | **r2** | **fa** | **cv** | **kd** | **ew** | **kw** | **cw** | **e_cwew** | **kn** | **sw** | **kr** | **cd1** | **cd3** | **cd2** | **cd4** | **m1** | **m2** | **rq1** | **rq2** | **en** | **av_ew** |
| --- | --- | --- | --- | --- | --- | --- | --- | --- | --- | --- | --- | --- | --- | --- | --- | --- | --- | --- | --- | --- | --- | --- | --- | --- | --- | --- | --- | --- | --- | --- |
| **yld** | r^a^ | **0.443** | -0.337 | 0.652 | 0.391 | 0.174 | 0.448 | 0.296 | 0.216 | 0.168 | -0.306 | -0.447 | 0.267 | 0.786 | 0.789 | 0.626 | 0.116 | 0.734 | 0.110 | 0.762 | 0.256 | 0.029 | 0.399 | 0.214 | 0.182 | 0.308 | 0.340 | 0.492 | 0.787 | 0.128 |
| **yld** | P | **0.000** | 0.000 | 0.000 | 0.000 | 0.054 | 0.000 | 0.001 | 0.017 | 0.064 | 0.001 | 0.000 | 0.003 | 0.000 | 0.000 | 0.000 | 0.201 | 0.000 | 0.227 | 0.000 | 0.004 | 0.751 | 0.000 | 0.017 | 0.044 | 0.001 | 0.000 | 0.000 | 0.000 | 0.158 |
| **cwew** | r | -0.068 | **0.218** | 0.023 | 0.080 | 0.089 | 0.072 | 0.088 | -0.083 | -0.126 | 0.035 | 0.084 | -0.234 | -0.022 | -0.105 | 0.208 | 0.446 | -0.231 | 0.226 | -0.234 | 0.245 | 0.225 | 0.229 | 0.214 | 0.106 | 0.068 | 0.192 | 0.201 | -0.167 | 0.054 |
| **cwew** | P | 0.452 | **0.016** | 0.799 | 0.382 | 0.326 | 0.430 | 0.335 | 0.360 | 0.165 | 0.697 | 0.358 | 0.009 | 0.809 | 0.246 | 0.021 | 0.000 | 0.010 | 0.012 | 0.009 | 0.006 | 0.012 | 0.011 | 0.017 | 0.245 | 0.458 | 0.034 | 0.026 | 0.065 | 0.555 |
| **l** | r | 0.483 | 0.103 | **0.708** | 0.137 | -0.065 | 0.161 | 0.051 | -0.120 | -0.136 | -0.322 | -0.321 | -0.137 | 0.772 | 0.736 | 0.717 | 0.302 | 0.609 | 0.229 | 0.807 | 0.261 | -0.058 | 0.310 | 0.109 | 0.251 | 0.253 | 0.343 | 0.411 | 0.502 | 0.297 |
| **l** | P | 0.000 | 0.259 | **0.000** | 0.131 | 0.472 | 0.075 | 0.573 | 0.186 | 0.134 | 0.000 | 0.000 | 0.131 | 0.000 | 0.000 | 0.000 | 0.001 | 0.000 | 0.011 | 0.000 | 0.004 | 0.522 | 0.001 | 0.231 | 0.005 | 0.005 | 0.000 | 0.000 | 0.000 | 0.001 |
| **ed1** | r | 0.409 | 0.102 | 0.097 | **0.668** | 0.871 | 0.965 | 0.887 | 0.686 | 0.628 | 0.170 | -0.149 | 0.540 | 0.635 | 0.628 | 0.530 | 0.137 | 0.438 | 0.350 | 0.145 | 0.811 | 0.726 | 0.798 | 0.719 | 0.643 | 0.632 | 0.810 | 0.763 | 0.102 | 0.559 |
| **ed1** | P | 0.000 | 0.261 | 0.285 | **0.000** | 0.000 | 0.000 | 0.000 | 0.000 | 0.000 | 0.061 | 0.099 | 0.000 | 0.000 | 0.000 | 0.000 | 0.131 | 0.000 | 0.000 | 0.110 | 0.000 | 0.000 | 0.000 | 0.000 | 0.000 | 0.000 | 0.000 | 0.000 | 0.262 | 0.000 |
| **ed3** | r | 0.309 | 0.059 | -0.054 | 0.870 | **0.629** | 0.824 | 0.955 | 0.682 | 0.701 | 0.528 | 0.024 | 0.517 | 0.432 | 0.440 | 0.324 | 0.025 | 0.294 | 0.282 | -0.056 | 0.700 | 0.853 | 0.606 | 0.735 | 0.577 | 0.511 | 0.659 | 0.530 | -0.099 | 0.528 |
| **ed3** | P | 0.001 | 0.519 | 0.554 | 0.000 | **0.000** | 0.000 | 0.000 | 0.000 | 0.000 | 0.000 | 0.791 | 0.000 | 0.000 | 0.000 | 0.000 | 0.787 | 0.001 | 0.002 | 0.540 | 0.000 | 0.000 | 0.000 | 0.000 | 0.000 | 0.000 | 0.000 | 0.000 | 0.278 | 0.000 |
| **ed2** | r | 0.443 | 0.109 | 0.086 | 0.970 | 0.822 | **0.649** | 0.894 | 0.641 | 0.567 | 0.050 | -0.215 | 0.559 | 0.664 | 0.662 | 0.540 | 0.125 | 0.464 | 0.367 | 0.192 | 0.720 | 0.634 | 0.809 | 0.726 | 0.548 | 0.639 | 0.744 | 0.801 | 0.161 | 0.534 |
| **ed2** | P | 0.000 | 0.230 | 0.343 | 0.000 | 0.000 | **0.000** | 0.000 | 0.000 | 0.000 | 0.581 | 0.017 | 0.000 | 0.000 | 0.000 | 0.000 | 0.169 | 0.000 | 0.000 | 0.033 | 0.000 | 0.000 | 0.000 | 0.000 | 0.000 | 0.000 | 0.000 | 0.000 | 0.076 | 0.000 |
| **ed4** | r | 0.336 | 0.071 | -0.039 | 0.866 | 0.957 | 0.860 | **0.542** | 0.618 | 0.615 | 0.340 | -0.092 | 0.541 | 0.551 | 0.558 | 0.425 | 0.055 | 0.362 | 0.370 | 0.052 | 0.678 | 0.772 | 0.699 | 0.807 | 0.538 | 0.570 | 0.662 | 0.638 | 0.006 | 0.556 |
| **ed4** | P | 0.000 | 0.438 | 0.665 | 0.000 | 0.000 | 0.000 | **0.000** | 0.000 | 0.000 | 0.000 | 0.312 | 0.000 | 0.000 | 0.000 | 0.000 | 0.543 | 0.000 | 0.000 | 0.568 | 0.000 | 0.000 | 0.000 | 0.000 | 0.000 | 0.000 | 0.000 | 0.000 | 0.950 | 0.000 |
| **r1** | r | 0.143 | -0.054 | -0.275 | 0.645 | 0.672 | 0.605 | 0.623 | **0.695** | 0.947 | 0.340 | -0.060 | 0.598 | 0.336 | 0.388 | 0.131 | -0.225 | 0.560 | -0.310 | 0.134 | 0.447 | 0.547 | 0.381 | 0.397 | 0.356 | 0.326 | 0.405 | 0.298 | -0.018 | 0.367 |
| **r1** | P | 0.114 | 0.557 | 0.002 | 0.000 | 0.000 | 0.000 | 0.000 | **0.000** | 0.000 | 0.000 | 0.512 | 0.000 | 0.000 | 0.000 | 0.149 | 0.012 | 0.000 | 0.001 | 0.140 | 0.000 | 0.000 | 0.000 | 0.000 | 0.000 | 0.000 | 0.000 | 0.001 | 0.844 | 0.000 |
| **r2** | r | 0.222 | -0.035 | -0.203 | 0.647 | 0.742 | 0.593 | 0.668 | 0.957 | **0.716** | 0.446 | 0.013 | 0.535 | 0.292 | 0.341 | 0.104 | -0.222 | 0.524 | -0.323 | 0.084 | 0.439 | 0.591 | 0.331 | 0.392 | 0.351 | 0.254 | 0.394 | 0.228 | -0.060 | 0.361 |
| **r2** | P | 0.014 | 0.704 | 0.024 | 0.000 | 0.000 | 0.000 | 0.000 | 0.000 | **0.000** | 0.000 | 0.884 | 0.000 | 0.001 | 0.000 | 0.255 | 0.014 | 0.000 | 0.000 | 0.357 | 0.000 | 0.000 | 0.000 | 0.000 | 0.000 | 0.005 | 0.000 | 0.011 | 0.512 | 0.000 |
| **fa** | r | 0.137 | 0.017 | -0.141 | 0.514 | 0.758 | 0.402 | 0.612 | 0.627 | 0.749 | **0.670** | 0.389 | 0.087 | -0.168 | -0.150 | -0.181 | -0.120 | -0.063 | -0.140 | -0.298 | 0.216 | 0.590 | -0.065 | 0.244 | 0.254 | 0.010 | 0.120 | -0.176 | -0.370 | 0.141 |
| **fa** | P | 0.130 | 0.851 | 0.121 | 0.000 | 0.000 | 0.000 | 0.000 | 0.000 | 0.000 | **0.000** | 0.000 | 0.340 | 0.064 | 0.097 | 0.045 | 0.186 | 0.487 | 0.122 | 0.001 | 0.016 | 0.000 | 0.476 | 0.007 | 0.005 | 0.911 | 0.185 | 0.052 | 0.000 | 0.121 |
| **cv** | r | -0.109 | -0.031 | -0.400 | 0.323 | 0.498 | 0.277 | 0.434 | 0.468 | 0.496 | 0.573 | **0.443** | -0.265 | -0.414 | -0.424 | -0.305 | -0.004 | -0.372 | -0.073 | -0.426 | -0.059 | 0.117 | -0.202 | -0.069 | 0.062 | -0.064 | -0.076 | -0.248 | -0.367 | -0.191 |
| **cv** | P | 0.232 | 0.737 | 0.000 | 0.000 | 0.000 | 0.002 | 0.000 | 0.000 | 0.000 | 0.000 | **0.000** | 0.003 | 0.000 | 0.000 | 0.001 | 0.962 | 0.000 | 0.421 | 0.000 | 0.518 | 0.199 | 0.025 | 0.448 | 0.495 | 0.483 | 0.402 | 0.006 | 0.000 | 0.035 |
| **kd** | r | 0.407 | -0.251 | -0.063 | 0.499 | 0.529 | 0.529 | 0.551 | 0.529 | 0.554 | 0.363 | 0.273 | **0.641** | 0.302 | 0.402 | -0.028 | -0.459 | 0.389 | 0.025 | 0.117 | 0.114 | 0.196 | 0.164 | 0.185 | 0.123 | 0.195 | 0.131 | 0.150 | 0.105 | 0.243 |
| **kd** | P | 0.000 | 0.005 | 0.489 | 0.000 | 0.000 | 0.000 | 0.000 | 0.000 | 0.000 | 0.000 | 0.002 | **0.000** | 0.001 | 0.000 | 0.762 | 0.000 | 0.000 | 0.786 | 0.196 | 0.211 | 0.030 | 0.070 | 0.041 | 0.175 | 0.030 | 0.149 | 0.097 | 0.250 | 0.007 |
| **ew** | r | 0.703 | 0.070 | 0.725 | 0.622 | 0.464 | 0.631 | 0.492 | 0.220 | 0.290 | 0.228 | -0.093 | 0.416 | **0.535** | 0.981 | 0.855 | 0.252 | 0.788 | 0.340 | 0.736 | 0.576 | 0.308 | 0.661 | 0.468 | 0.443 | 0.481 | 0.650 | 0.708 | 0.460 | 0.568 |
| **ew** | P | 0.000 | 0.439 | 0.000 | 0.000 | 0.000 | 0.000 | 0.000 | 0.014 | 0.001 | 0.011 | 0.306 | 0.000 | **0.000** | 0.000 | 0.000 | 0.005 | 0.000 | 0.000 | 0.000 | 0.000 | 0.001 | 0.000 | 0.000 | 0.000 | 0.000 | 0.000 | 0.000 | 0.000 | 0.000 |
| **kw** | r | 0.706 | -0.024 | 0.667 | 0.624 | 0.493 | 0.637 | 0.522 | 0.283 | 0.357 | 0.271 | -0.052 | 0.525 | 0.980 | **0.521** | 0.740 | 0.067 | 0.841 | 0.280 | 0.767 | 0.497 | 0.266 | 0.586 | 0.415 | 0.423 | 0.476 | 0.570 | 0.630 | 0.449 | 0.571 |
| **kw** | P | 0.000 | 0.789 | 0.000 | 0.000 | 0.000 | 0.000 | 0.000 | 0.002 | 0.000 | 0.002 | 0.565 | 0.000 | 0.000 | **0.000** | 0.000 | 0.463 | 0.000 | 0.002 | 0.000 | 0.000 | 0.003 | 0.000 | 0.000 | 0.000 | 0.000 | 0.000 | 0.000 | 0.000 | 0.000 |
| **cw** | r | 0.574 | 0.285 | 0.738 | 0.510 | 0.314 | 0.509 | 0.336 | 0.033 | 0.081 | 0.087 | -0.175 | 0.084 | 0.877 | 0.763 | **0.639** | 0.706 | 0.490 | 0.437 | 0.507 | 0.678 | 0.362 | 0.736 | 0.523 | 0.411 | 0.401 | 0.743 | 0.781 | 0.399 | 0.449 |
| **cw** | P | 0.000 | 0.001 | 0.000 | 0.000 | 0.000 | 0.000 | 0.000 | 0.721 | 0.371 | 0.339 | 0.054 | 0.356 | 0.000 | 0.000 | **0.000** | 0.000 | 0.000 | 0.000 | 0.000 | 0.000 | 0.000 | 0.000 | 0.000 | 0.000 | 0.000 | 0.000 | 0.000 | 0.000 | 0.000 |
| **e_cwew** | r | 0.094 | 0.536 | 0.408 | 0.111 | -0.060 | 0.094 | -0.061 | -0.251 | -0.257 | -0.156 | -0.186 | -0.452 | 0.274 | 0.080 | 0.691 | **0.770** | -0.146 | 0.380 | -0.044 | 0.477 | 0.265 | 0.488 | 0.358 | 0.166 | 0.114 | 0.501 | 0.514 | 0.145 | 0.063 |
| **e_cwew** | P | 0.302 | 0.000 | 0.000 | 0.222 | 0.513 | 0.303 | 0.501 | 0.005 | 0.004 | 0.085 | 0.039 | 0.000 | 0.002 | 0.377 | 0.000 | **0.000** | 0.107 | 0.000 | 0.627 | 0.000 | 0.003 | 0.000 | 0.000 | 0.066 | 0.209 | 0.000 | 0.000 | 0.110 | 0.486 |
| **m\c** |  | **yld** | **cwew** | **l** | **ed1** | **ed3** | **ed2** | **ed4** | **r1** | **r2** | **fa** | **cv** | **kd** | **ew** | **kw** | **cw** | **e_cwew** | **kn** | **sw** | **kr** | **cd1** | **cd3** | **cd2** | **cd4** | **m1** | **m2** | **rq1** | **rq2** | **en** | **av_ew** |
| **kn** | r | 0.534 | -0.045 | 0.493 | 0.421 | 0.394 | 0.429 | 0.389 | 0.486 | 0.564 | 0.394 | 0.089 | 0.458 | 0.735 | 0.779 | 0.503 | -0.063 | **0.587** | -0.275 | 0.868 | 0.264 | 0.131 | 0.324 | 0.188 | 0.259 | 0.299 | 0.313 | 0.360 | 0.465 | 0.378 |
| **kn** | P | 0.000 | 0.623 | 0.000 | 0.000 | 0.000 | 0.000 | 0.000 | 0.000 | 0.000 | 0.000 | 0.329 | 0.000 | 0.000 | 0.000 | 0.000 | 0.491 | **0.000** | 0.002 | 0.000 | 0.003 | 0.150 | 0.000 | 0.037 | 0.004 | 0.001 | 0.000 | 0.000 | 0.000 | 0.000 |
| **sw** | r | 0.374 | 0.041 | 0.358 | 0.406 | 0.235 | 0.419 | 0.284 | -0.237 | -0.220 | -0.097 | -0.182 | 0.177 | 0.512 | 0.482 | 0.495 | 0.225 | -0.161 | **0.640** | -0.169 | 0.410 | 0.254 | 0.469 | 0.407 | 0.282 | 0.312 | 0.448 | 0.482 | -0.005 | 0.325 |
| **sw** | P | 0.000 | 0.653 | 0.000 | 0.000 | 0.009 | 0.000 | 0.001 | 0.008 | 0.015 | 0.286 | 0.044 | 0.051 | 0.000 | 0.000 | 0.000 | 0.013 | 0.076 | **0.000** | 0.062 | 0.000 | 0.005 | 0.000 | 0.000 | 0.002 | 0.000 | 0.000 | 0.000 | 0.959 | 0.000 |
| **kr** | r | 0.478 | -0.061 | 0.779 | -0.032 | -0.137 | -0.003 | -0.121 | -0.157 | -0.069 | -0.101 | -0.302 | 0.069 | 0.610 | 0.609 | 0.508 | 0.126 | 0.719 | -0.044 | **0.681** | 0.085 | -0.162 | 0.174 | -0.024 | 0.157 | 0.215 | 0.162 | 0.277 | 0.583 | 0.226 |
| **kr** | P | 0.000 | 0.502 | 0.000 | 0.722 | 0.131 | 0.969 | 0.184 | 0.082 | 0.447 | 0.264 | 0.001 | 0.450 | 0.000 | 0.000 | 0.000 | 0.167 | 0.000 | 0.627 | **0.000** | 0.349 | 0.073 | 0.055 | 0.791 | 0.083 | 0.017 | 0.073 | 0.002 | 0.000 | 0.012 |
| **cd1** | r | 0.279 | 0.204 | 0.245 | 0.824 | 0.655 | 0.736 | 0.596 | 0.481 | 0.482 | 0.427 | 0.137 | 0.129 | 0.576 | 0.515 | 0.623 | 0.400 | 0.310 | 0.383 | -0.013 | **0.727** | 0.804 | 0.876 | 0.746 | 0.765 | 0.602 | 0.945 | 0.777 | 0.028 | 0.533 |
| **cd1** | P | 0.002 | 0.023 | 0.006 | 0.000 | 0.000 | 0.000 | 0.000 | 0.000 | 0.000 | 0.000 | 0.130 | 0.156 | 0.000 | 0.000 | 0.000 | 0.000 | 0.001 | 0.000 | 0.886 | **0.000** | 0.000 | 0.000 | 0.000 | 0.000 | 0.000 | 0.000 | 0.000 | 0.758 | 0.000 |
| **cd3** | r | 0.202 | 0.163 | -0.038 | 0.757 | 0.880 | 0.665 | 0.792 | 0.612 | 0.678 | 0.790 | 0.443 | 0.299 | 0.372 | 0.362 | 0.331 | 0.114 | 0.312 | 0.146 | -0.177 | 0.721 | **0.682** | 0.653 | 0.832 | 0.676 | 0.525 | 0.716 | 0.521 | -0.196 | 0.495 |
| **cd3** | P | 0.025 | 0.072 | 0.673 | 0.000 | 0.000 | 0.000 | 0.000 | 0.000 | 0.000 | 0.000 | 0.000 | 0.001 | 0.000 | 0.000 | 0.000 | 0.210 | 0.000 | 0.107 | 0.050 | 0.000 | **0.000** | 0.000 | 0.000 | 0.000 | 0.000 | 0.000 | 0.000 | 0.030 | 0.000 |
| **cd2** | r | 0.362 | 0.248 | 0.313 | 0.786 | 0.527 | 0.798 | 0.570 | 0.327 | 0.297 | 0.130 | -0.001 | 0.126 | 0.629 | 0.562 | 0.682 | 0.444 | 0.293 | 0.492 | 0.069 | 0.867 | 0.531 | **0.683** | 0.830 | 0.618 | 0.678 | 0.869 | 0.913 | 0.161 | 0.500 |
| **cd2** | P | 0.000 | 0.006 | 0.000 | 0.000 | 0.000 | 0.000 | 0.000 | 0.000 | 0.001 | 0.153 | 0.994 | 0.163 | 0.000 | 0.000 | 0.000 | 0.000 | 0.001 | 0.000 | 0.450 | 0.000 | 0.000 | **0.000** | 0.000 | 0.000 | 0.000 | 0.000 | 0.000 | 0.075 | 0.000 |
| **cd4** | r | 0.263 | 0.220 | 0.050 | 0.730 | 0.783 | 0.712 | 0.825 | 0.446 | 0.481 | 0.516 | 0.271 | 0.278 | 0.441 | 0.421 | 0.413 | 0.182 | 0.282 | 0.279 | -0.122 | 0.629 | 0.863 | 0.639 | **0.535** | 0.583 | 0.640 | 0.712 | 0.728 | -0.030 | 0.502 |
| **cd4** | P | 0.003 | 0.014 | 0.584 | 0.000 | 0.000 | 0.000 | 0.000 | 0.000 | 0.000 | 0.000 | 0.002 | 0.002 | 0.000 | 0.000 | 0.000 | 0.044 | 0.002 | 0.002 | 0.180 | 0.000 | 0.000 | 0.000 | **0.000** | 0.000 | 0.000 | 0.000 | 0.000 | 0.745 | 0.000 |
| **m1** | r | 0.203 | 0.053 | 0.138 | 0.646 | 0.519 | 0.544 | 0.438 | 0.408 | 0.409 | 0.410 | 0.120 | 0.114 | 0.381 | 0.370 | 0.340 | 0.112 | 0.230 | 0.266 | -0.039 | 0.847 | 0.629 | 0.679 | 0.508 | **0.673** | 0.839 | 0.789 | 0.596 | -0.049 | 0.515 |
| **m1** | P | 0.025 | 0.558 | 0.128 | 0.000 | 0.000 | 0.000 | 0.000 | 0.000 | 0.000 | 0.000 | 0.186 | 0.210 | 0.000 | 0.000 | 0.000 | 0.219 | 0.011 | 0.003 | 0.672 | 0.000 | 0.000 | 0.000 | 0.000 | **0.000** | 0.000 | 0.000 | 0.000 | 0.588 | 0.000 |
| **m2** | r | 0.252 | 0.071 | 0.165 | 0.568 | 0.381 | 0.562 | 0.385 | 0.272 | 0.257 | 0.156 | 0.015 | 0.142 | 0.387 | 0.387 | 0.319 | 0.059 | 0.233 | 0.300 | 0.049 | 0.671 | 0.427 | 0.742 | 0.473 | 0.840 | **0.665** | 0.677 | 0.712 | 0.049 | 0.468 |
| **m2** | P | 0.005 | 0.433 | 0.069 | 0.000 | 0.000 | 0.000 | 0.000 | 0.002 | 0.004 | 0.086 | 0.872 | 0.117 | 0.000 | 0.000 | 0.000 | 0.516 | 0.009 | 0.001 | 0.594 | 0.000 | 0.000 | 0.000 | 0.000 | 0.000 | **0.000** | 0.000 | 0.000 | 0.592 | 0.000 |
| **rq1** | r | 0.358 | 0.214 | 0.308 | 0.802 | 0.624 | 0.723 | 0.582 | 0.395 | 0.398 | 0.388 | 0.099 | 0.107 | 0.619 | 0.548 | 0.683 | 0.450 | 0.315 | 0.432 | 0.048 | 0.954 | 0.693 | 0.844 | 0.643 | 0.822 | 0.659 | **0.670** | 0.868 | 0.082 | 0.560 |
| **rq1** | P | 0.000 | 0.017 | 0.001 | 0.000 | 0.000 | 0.000 | 0.000 | 0.000 | 0.000 | 0.000 | 0.274 | 0.241 | 0.000 | 0.000 | 0.000 | 0.000 | 0.000 | 0.000 | 0.600 | 0.000 | 0.000 | 0.000 | 0.000 | 0.000 | 0.000 | **0.000** | 0.000 | 0.365 | 0.000 |
| **rq2** | r | 0.406 | 0.266 | 0.346 | 0.754 | 0.509 | 0.780 | 0.561 | 0.264 | 0.235 | 0.120 | -0.016 | 0.114 | 0.651 | 0.572 | 0.729 | 0.492 | 0.312 | 0.485 | 0.124 | 0.793 | 0.508 | 0.933 | 0.644 | 0.612 | 0.702 | 0.855 | **0.662** | 0.256 | 0.471 |
| **rq2** | P | 0.000 | 0.003 | 0.000 | 0.000 | 0.000 | 0.000 | 0.000 | 0.003 | 0.009 | 0.186 | 0.865 | 0.209 | 0.000 | 0.000 | 0.000 | 0.000 | 0.000 | 0.000 | 0.174 | 0.000 | 0.000 | 0.000 | 0.000 | 0.000 | 0.000 | 0.000 | **0.000** | 0.004 | 0.000 |
| **en** | r | 0.645 | 0.001 | 0.071 | -0.062 | -0.080 | -0.022 | -0.050 | -0.105 | -0.076 | -0.136 | -0.150 | 0.068 | 0.044 | 0.037 | 0.052 | 0.025 | 0.005 | 0.064 | 0.102 | -0.152 | -0.138 | -0.069 | -0.083 | -0.165 | -0.098 | -0.086 | -0.015 | **0.354** | -0.372 |
| **en** | P | 0.000 | 0.995 | 0.436 | 0.497 | 0.378 | 0.812 | 0.583 | 0.248 | 0.406 | 0.135 | 0.097 | 0.455 | 0.631 | 0.683 | 0.568 | 0.780 | 0.958 | 0.482 | 0.262 | 0.094 | 0.129 | 0.448 | 0.359 | 0.069 | 0.280 | 0.345 | 0.868 | **0.000** | 0.000 |
| **av_ew** | r | 0.149 | 0.023 | 0.484 | 0.509 | 0.405 | 0.485 | 0.404 | 0.277 | 0.312 | 0.299 | 0.080 | 0.280 | 0.718 | 0.716 | 0.599 | 0.148 | 0.571 | 0.332 | 0.397 | 0.516 | 0.380 | 0.496 | 0.389 | 0.403 | 0.370 | 0.510 | 0.482 | -0.591 | **0.292** |
| **av_ew** | P | 0.100 | 0.801 | 0.000 | 0.000 | 0.000 | 0.000 | 0.000 | 0.002 | 0.000 | 0.001 | 0.377 | 0.002 | 0.000 | 0.000 | 0.000 | 0.103 | 0.000 | 0.000 | 0.000 | 0.000 | 0.000 | 0.000 | 0.000 | 0.000 | 0.000 | 0.000 | 0.000 | 0.000 | **0.001** |

^a^ squares diagonal indicates the Pearson coefficient correlation (r) for each trait between the two environments and respective P-value (very strong correlation (0.90 to 1.00) in black; strong (0.70 to 0.90) in dark grey and moderate (0.65 to 0.7) in light grey)

Traits measured: yld –yield; cwew - cob/ear weight at harvest; en - ears number; av_ew - average 20 ears weight; l – ear length; ed 1 to 4 - ear diameter 1 to 4; cd1 to 4 - cob diameter 1 to 3; kd - kernel dept; m1, m2 - medulla 1 and 2; rq1, rq2 -rachis 1 and 2; ew -ear weight; cw -cob weight; sw -thousand kernel weight; kw - kernel weight; e_cwew - cob/ear weight per ear; r1, r2 - kernel-row number 1 and 2; fa - fasciation; cv - ear convulsion; kn - kernel number; nc - kernel per row.
