# Supplementary material for: Genetic Architecture of Ear Fasciation in Maize (Zea mays) under QTL Scrutiny
Source: PLoS One. 2015 Apr 29;10(4):e0124543. doi: 10.1371/journal.pone.0124543 (PMC4414412; doi:10.1371/journal.pone.0124543)
Supplement: S2 Table — a PC loading scales correlation in absolute values: very weak: 0.00 to 0.20; weak: 0.20 to 0.40; moderate: 0.40 to 0.70; strong (in grey): 0.70 to 0.90; very strong (in black): 0.90 to 1.00. Levels of significance: ns non-significant value; * significant at P < 0.05; ** significant at P < 0.01; *** significant at P < 0.0 (DOCX) [file pone.0124543.s002.docx]

**Table S2** Component loadings for the first three Principal Components (PC) of 29 ear fasciation and related traits in the maize F2 (PB260xPB266) in two environments

|  | **Coimbra** | | | | | | **Montemor** | | | | | |
| --- | --- | --- | --- | --- | --- | --- | --- | --- | --- | --- | --- | --- |
| **Trait^a^** | **PC1** |  | **PC2** |  | **PC3** |  | **PC1** |  | **PC2** |  | **PC3** |  |
| Eigenvalue | 12.65 |  | 5.11 |  | 3.69 |  | 12.45 |  | 4.96 |  | 3.21 |  |
| % of explained variance | 43.61 |  | 17.63 |  | 12.71 |  | 42.93 |  | 17.09 |  | 11.06 |  |
| Grain yield | 0.55 | *** | **0.70** | *** | 0.19 | * | 0.51 | *** | 0.38 | *** | 0.44 | *** |
| Cob/ear weight at harvest | 0.10 | ns | -0.24 | ** | -0.52 | *** | 0.14 | ns | 0.16 | ns | -0.43 | *** |
| Ears number | 0.21 | * | **0.71** | *** | 0.08 | ns | -0.08 | ns | 0.18 | * | 0.21 | * |
| Average Ear weight at harvest | 0.65 | *** | -0.12 | ns | 0.02 | ns | 0.65 | *** | 0.22 | * | 0.15 | ns |
| Ear length | 0.42 | *** | **0.75** | *** | -0.20 | * | 0.32 | *** | **0.82** | *** | 0.23 | * |
| Ear diameter 1 | **0.92** | *** | -0.22 | * | 0.13 | ns | **0.93** | *** | -0.18 | * | -0.07 | ns |
| Ear diameter 3 | **0.79** | *** | -0.49 | *** | 0.22 | * | **0.83** | *** | -0.44 | *** | 0.05 | ns |
| Ear diameter 2 | **0.90** | *** | -0.13 | ns | 0.13 | ns | **0.90** | *** | -0.13 | ns | -0.03 | ns |
| Ear diameter 4 | **0.84** | *** | -0.33 | *** | 0.17 | ns | **0.82** | *** | -0.37 | *** | 0.05 | ns |
| Kernel-row number 1 | 0.58 | *** | -0.29 | ** | 0.63 | *** | 0.59 | *** | -0.61 | *** | 0.23 | * |
| Kernel-row number 2 | 0.54 | *** | -0.35 | *** | 0.63 | *** | 0.63 | *** | -0.59 | *** | 0.31 | ** |
| Fasciation | 0.11 | ns | -0.65 | *** | 0.24 | ** | 0.55 | *** | -0.56 | *** | 0.18 | * |
| Convulsion | -0.24 | ** | -0.50 | *** | -0.12 | ns | 0.24 | ** | -0.66 | *** | 0.06 | ns |
| Kernel dept | 0.39 | *** | -0.09 | ns | 0.65 | *** | 0.45 | *** | -0.30 | ** | 0.58 | *** |
| Ear weight | **0.83** | *** | 0.50 | *** | 0.03 | ns | **0.79** | *** | 0.49 | *** | 0.33 | *** |
| Kernel weight | **0.80** | *** | 0.50 | *** | 0.18 | * | **0.77** | *** | 0.40 | *** | 0.45 | *** |
| Cob weight | **0.75** | *** | 0.41 | *** | -0.39 | *** | 0.69 | *** | 0.64 | *** | -0.02 | ns |
| Cob/ear weight per ear | 0.29 | ** | 0.08 | ns | **-0.78** | *** | 0.22 | * | 0.54 | *** | -0.53 | *** |
| Kernel number | 0.58 | *** | 0.52 | *** | 0.53 | *** | 0.58 | *** | 0.15 | ns | 0.67 | *** |
| Thousand kernel weight | 0.38 | *** | -0.04 | ns | -0.60 | *** | 0.41 | *** | 0.41 | *** | -0.24 | ** |
| Number of kernel per row | 0.37 | *** | **0.80** | *** | 0.26 | ** | 0.17 | ns | 0.65 | *** | 0.59 | *** |
| Cob diameter 1 | **0.86** | *** | -0.23 | * | -0.27 | ** | **0.87** | *** | 0.03 | ns | -0.35 | *** |
| Cob diameter 3 | **0.72** | *** | -0.57 | *** | -0.05 | ns | **0.79** | *** | -0.41 | *** | -0.15 | ns |
| Cob diameter 2 | **0.88** | *** | -0.05 | ns | -0.30 | ** | **0.83** | *** | 0.23 | * | -0.37 | *** |
| Cob diameter 4 | **0.79** | *** | -0.32 | *** | -0.20 | * | **0.78** | *** | -0.21 | * | -0.20 | * |
| Medulla 1 | **0.72** | *** | -0.23 | * | -0.13 | ns | **0.71** | *** | -0.07 | ns | -0.32 | *** |
| Medulla 2 | **0.72** | *** | -0.09 | ns | -0.11 | ns | 0.64 | *** | 0.07 | ns | -0.29 | ** |
| Rachis 1 | **0.89** | *** | -0.12 | ns | -0.28 | ** | **0.87** | *** | 0.12 | ns | -0.35 | *** |
| Rachis 2 | **0.86** | *** | 0.10 | ns | -0.32 | *** | **0.82** | *** | 0.28 | ** | -0.34 | *** |

^a^ PC loading scales correlation in absolute values: very weak: 0.00 to 0.20; weak: 0.20 to 0.40; moderate: 0.40 to 0.70; strong (in grey): 0.70 to 0.90; very strong (in black): 0.90 to 1.00.

Levels of significance: ns non-significant value; * significant at P < 0.05; ** significant at P < 0.01; *** significant at P < 0.001
